# Declined RTN3 stabilizes DHCR7 to induce cholesterol-dependent tumor progression and MEK inhibitors insensitivity in thyroid cancer

**DOI:** 10.1038/s41419-026-08538-y

**Published:** 2026-03-11

**Authors:** Anwen Ren, Nan Feng, Tinglin Yang, Zimei Tang, Huan Liu, Yi Li, Qingyi Hu, Zihan Xi, Jiaqing Zhu, Jun Zhou, Jie Ming, Nan Liu, Tao Huang, Ming Xu

**Affiliations:** 1 Department of Breast and Thyroid Surgery, Union Hospital, Tongji Medical College, Huazhong University of Science and Technology, Wuhan, China; 2 Department of Thyroid Surgery, Qilu Hospital of Shandong University, Jinan, China; 3 Department of Head-Neck and Thyroid Surgery, The First Affiliated Hospital of USTC, Division of Life Sciences and Medicine, University of Science and Technology of China, Hefei, China; 4 Department of Head-Neck and Thyroid Surgery, Anhui Provincial Cancer Hospital, Hefei, China; 5 First Clinical College, Tongji Medical College, Huazhong University of Science and Technology, Wuhan, China

**Keywords:** Thyroid cancer, Cancer metabolism

## Abstract

Mechanism underlying thyroid cancer progression and treatment resistance remains an unsolved problem in clinical practice. Endoplasmic reticulum (ER) proteins modulate cell biosynthesis and mediate tumor progression, among which Reticulon 3 (RTN3) is verified to play important roles in cancers. However, its effect in thyroid cancer has not been clarified. Meanwhile, cholesterol is found to contribute to proliferation and drug resistance in many tumors. As ER is the primary site of cholesterol synthesis, we aimed to study how RTN3 regulates cholesterol concentration and influences tumor progression and sensitivity to MEK inhibitors in thyroid cancer. This study found that RTN3 is low-expressed in thyroid cancer, and is related to poor prognosis and insensitivity to MEK inhibitors. It binds to a cholesterol synthesis enzyme DHCR7 and promotes its ubiquitination. Downregulation of RTN3 lead to stabilization of DHCR7 and elevate cholesterol concentration, activating EGFR/ERK pathway and contributes to progression of thyroid cancer, which can be rescued by HMG-CoA reductase inhibitor Simvastatin. We identified RTN3 as a tumor suppressor and a biomarker of sensitivity to MEK inhibitors and verified the role of cholesterol in drug resistance. The combination of statins provides a novel therapeutic method in patients resistant to MEK inhibitors.

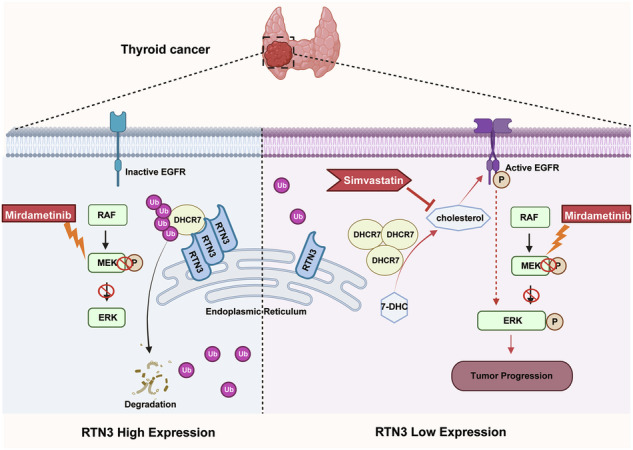

## Introduction

Thyroid cancer is the most prevalent malignancy in endocrine system and has the 7th highest incidence of malignant tumors globally [1]. Following standard treatment, including surgery, thyroid-stimulating hormone suppression therapy, radioactive iodine therapy and target therapy (like BRAF and MEK inhibitors), the 10-year overall survival rate of patients can exceed 90% [2, 3]. However, approximately 5–15% of cases develop treatment resistance, manifesting as adverse clinical outcomes such as local recurrence, radioactive iodine refractoriness, distant metastasis, resistance to target drugs, and dedifferentiation, which significantly impact patient prognosis [4]. Current research urgently needs to delve deeper into the molecular mechanisms underlying the pathogenesis and progression of thyroid cancer. By identifying key signaling pathways and biomarkers, novel therapeutic targets can be discovered to improve the progression-free survival of patients with refractory disease.

Endoplasmic reticulum (ER) proteins critically contribute to tumorigenesis by modulating cancer cell proliferation, apoptosis resistance, and microenvironment remodeling [5, 6]. Besides, many of them were demonstrated to be related to cancer drug resistance [7, 8]. Reticulon 3 (RTN3) localizes to the luminal ER membrane via its C-terminal reticulon homology domain. It not only maintains ER homeostasis by facilitating tubular network formation, modulating autophagy, and regulating Ca²⁺ flux, but has also been implicated in oncogenesis [9]. However, the roles of RTN3 in thyroid carcinogenesis and drug sensitivity remain unrevealed, and its molecular mechanisms for modulating malignant phenotypes are poorly understood.

The role of metabolic reprogramming in tumorigenesis has gained increasing recognition in recent years [10]. Among all metabolites related to lipid synthesis, cholesterol plays a crucial role in tumor proliferation and growth. On one hand, cholesterol serves as a core component of cellular and organellar membranes, and thus rapidly growing cancer cells are sensitive to its concentration [11]. On the other hand, cholesterol is a second messenger itself, participating in several signaling pathways [12, 13]. In lung cancer [14], breast cancer [15], pancreatic cancer [16] and hepatocellular cancer [17], cholesterol was reported to be involved in both chemotherapy and target therapy resistance [18] and cholesterol-lowering agents have emerged as therapeutic targets for overcoming tumor drug resistance [19]. Especially, Wang et al. found that altered cholesterol metabolism contributes to the tumor adaptive response upon targeted MAPK pathway inhibitors [20], which was also the main target of thyroid cancer therapy. Although associations between cholesterol and thyroid cancer progression [21, 22] and drug resistance [23] have been reported, the specific molecular mechanisms through which cholesterol influences tumor progression remain poorly defined.

Statins, as HMG-CoA reductase inhibitors, suppress cholesterol synthesis via the mevalonate pathway. Patients using statins exhibit improved prognosis across multiple cancers including breast, lung, prostate, and ovarian carcinomas [24–26]. Furthermore, statins show synergistic antitumor effects with several anti-tumor drugs like cisplatin, gefitinib and dabrafenib [27–29]. However, the therapeutic potential of statin-MEK inhibitor combinations remains underexplored, particularly in thyroid cancer. Notably, ER serves as the primary site for cholesterol biosynthesis, and it is the place where the reaction catalyzed by HMG-CoA reductase occurs. Therefore, it is worth studying that whether molecules on the ER membrane like RTN3 regulate tumor progression and drug resistance by modulating cholesterol biosynthesis.

This study revealed that low expression of RTN3 enhances progression and reduces sensitivity to Mirdametinib in thyroid cancer. RTN3 facilitates the degradation of the cholesterol synthesis enzyme DHCR7 by promoting its ubiquitination. Consequently, low RTN3 expression leads to increased DHCR7 stability, elevated cholesterol levels, and subsequent activation of the EGFR/ERK pathway. Therefore, in thyroid cancer with low RTN3 expression, the combination of Mirdametinib and Simvastatin effectively suppresses tumor progression. This study identifies RTN3 as a potential prognostic biomarker for thyroid cancer progression and Mirdametinib sensitivity, and provides a novel combination therapeutic strategy.

## Results

### Low expression of RTN3 is correlated with progression of thyroid cancer

**Fig. 1:**
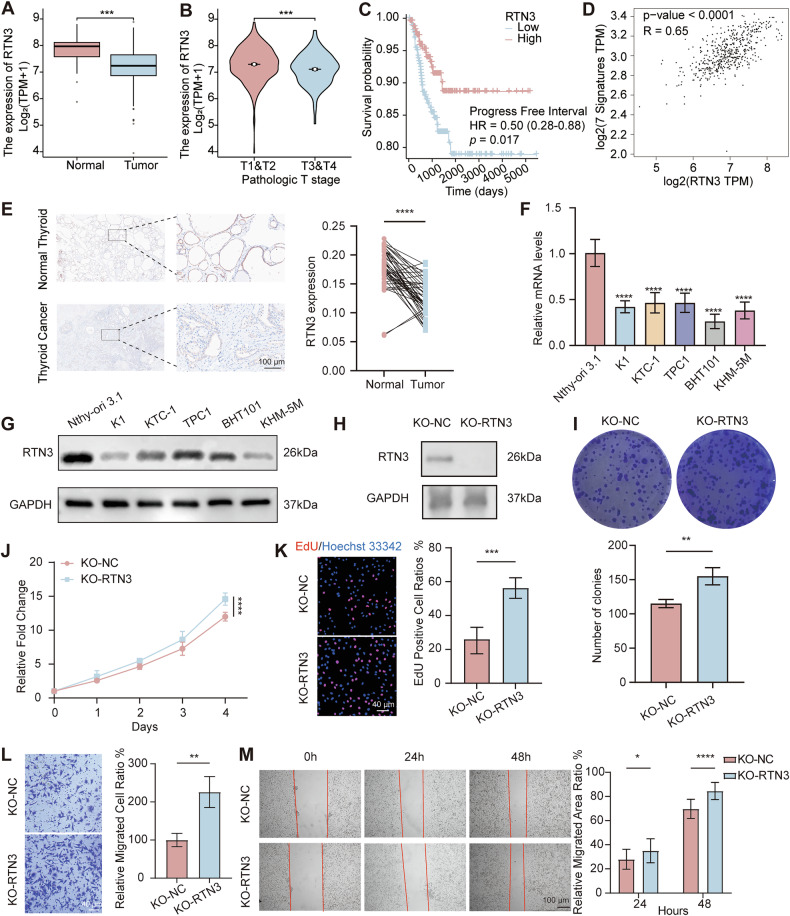
RTN3 is down-regulated in thyroid cancer and its low expression is associated with poor prognosis. **A** mRNA level of RTN3 in thyroid cancer and normal tissues from TCGA database. **B** Relationship between RTN3 expression and pathologic T stage in thyroid cancer patients from TCGA database. **C** Kaplan–Meier survival analysis of progress free survival in patients with low or high RTN3 expression from TCGA-THCA cohort. **D** Association of RTN3 and gene set of thyroid cancer dedifferentiation (FOXE1, NKX2-1, NIS, PAX8, TG, TPO and TSHR). **E** Representative IHC staining images of RTN3 (left) and relative expression levels (right) in 45 pairs of thyroid cancer and adjacent normal tissues (scale bar, 100 μm). **F**, **G** qRT-PCR (**F**) and WB (**G**) detected mRNA and protein expression levels of RTN3 in normal thyroid and thyroid cancer cell lines. **H**, Validation of RTN3 knockout by WB. **I–K** Cell proliferation ability after RTN3 knockout determined by colony-formation assays (**I**)**,** CCK-8 assays (**J**) and EdU assays (**K**) with representative images in the left (scale bar, 40 μm) and the quantification of EdU positive cell ratios in the right, *n* = 3. **L**, **M** Cell migration ability after RTN3 knockout determined by transwell assays (**L**) (scale bar, 40 μm) and wound healing assays (**M**) (scale bar, 100 μm), with representative images in the left and the quantification graphs in the right, *n* = 3. *: *P* < 0.05, **: *P* < 0.01, ***: *P* < 0.001, ****: *P* < 0.0001.

It is reported that RTN3 expression was remarkedly reduced in hepatocellular cancer, but its role in other cancers is unrevealed yet. Analysis of TCGA-THCA dataset demonstrated significant low-expression of RTN3 in thyroid carcinoma tissues compared to normal thyroid tissues (Fig. 1A) and in higher stages patients compared to lower stages (Fig. 1B). Survival analysis demonstrated that patients with low RTN3 expression had poorer progression-free survival (Fig. 1C). As dedifferentiation is a key feature of thyroid cancer progression, we evaluated RTN3’s correlation with dedifferentiation markers FOXE1, NKX2-1, NIS, PAX8, TG, TPO and TSHR [30] and identified a significant positive association between RTN3 and the gene set (Fig. 1D), indicating RTN3 is related to better differentiation. IHC staining of 45 pairs of thyroid carcinoma tissues and adjacent noncancerous tissues confirmed decreased RTN3 expression in thyroid tumor (Fig. 1E). Using the median expression as the cutoff, patients were divided into high and low RTN3 groups. Analysis revealed that the low-expression group had a significantly higher lymph node ratio and higher N stage—a clinically important prognostic factor for recurrence (Supplementary Table S1). Consistent with clinical findings, thyroid cancer cell lines K1, KTC-1, TPC1, BHT101, KHM-5M showed reduced RTN3 mRNA (Fig. 1F) and protein (Fig. 1G) levels versus normal thyroid cells Nthy-ori 3.1. The above results indicate that RTN3 expression is low in thyroid carcinoma, and this low expression is associated with a poor prognosis.

**Fig. 2:**
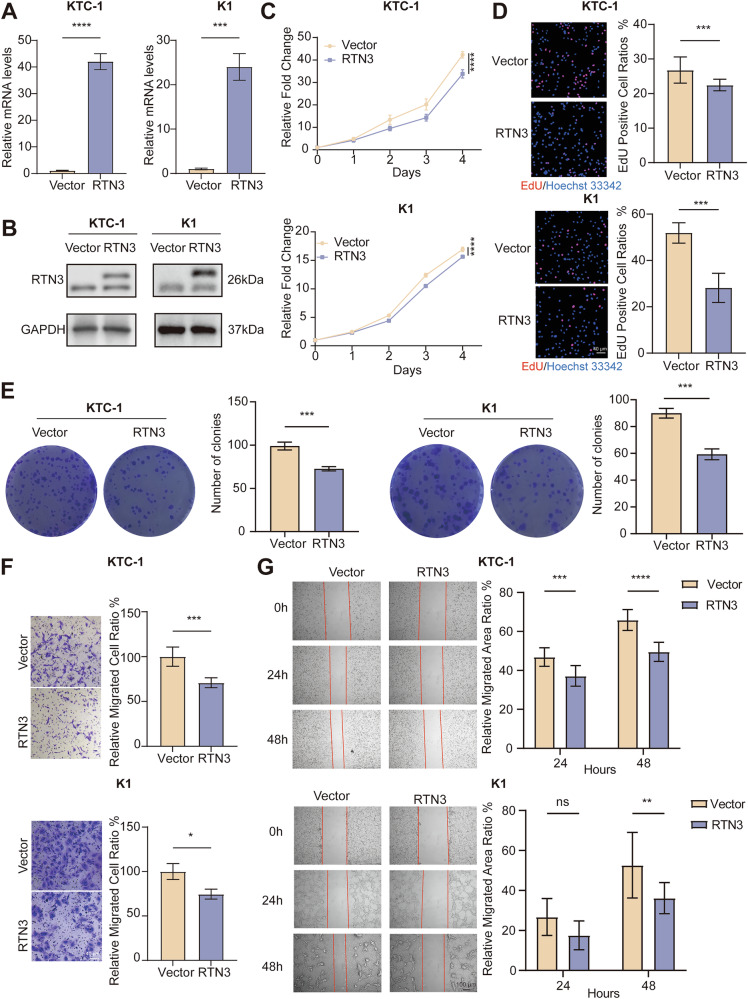
Overexpression of RTN3 inhibits proliferation and migration of thyroid cancer cells. **A**, **B** Validation of the overexpression efficiency of RTN3 plasmids by qRT-PCR (**A**) and WB (**B**) in KTC-1 and K1 cells. **C–E** Cell proliferation ability of RTN3 overexpression cells determined by CCK-8 assays (**C**), EdU assays (**D**) with representative images in the left (scale bar, 40 μm) and the quantification of EdU positive cell ratios in the right, and colony-formation assays (**E**), *n* = 3. **F**, **G** Cell migration ability of RTN3 overexpression cells determined by transwell assays (**F**) (scale bar, 40 μm) and wound healing assays (**G**) (scale bar, 100 μm), with representative images in the left and the quantification graphs in the right, *n* = 3. *: *P* < 0.05, **: *P* < 0.01, ***: *P* < 0.001, ****: *P* < 0.0001, ns: *P* ≥ 0.05.

To further explore the biological functions of RTN3, we constructed CRISPR/Cas9-mediated RTN3 knockout in KTC-1 cells. Knockout of RTN3 was confirmed by WB analysis (Fig. 1H). Colony-formation (Fig. 1I), CCK-8 (Fig. 1J), and EdU (Fig. 1K) assays demonstrated that RTN3 knockout promoted the proliferation ability of thyroid cancer. Transwell (Fig. 1L) and wound healing (Fig. 1M) assays indicated enhanced migration capacities in knockout cells. Besides, we performed siRNA-mediated knockdown and overexpression of RTN3 (using pCMV-RTN3-3xFlag-Neo plasmid) in KTC-1 and K1 cells, with efficiency validated by qRT-PCR (Supplementary Fig. S1A and Fig. 2A) and WB (Supplementary Fig. S1B and Fig. 2B). Functional experiments demonstrated when RTN3 is downregulated, proliferation and migration ability of cells was enhanced (Supplementary Fig. S1C-G) and *vice visa* (Fig. 2C-G). Collectively, these results demonstrated that RTN3 suppresses thyroid cancer progression in vitro.

### Identification of DHCR7 as a RTN3-interacting protein

**Fig. 3:**
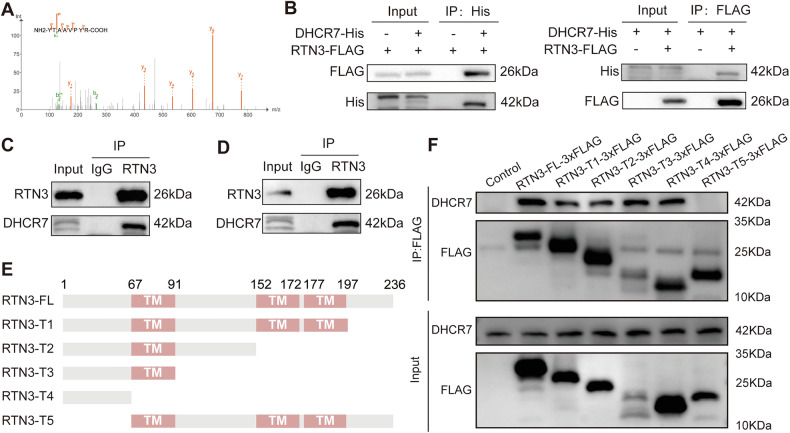
RTN3 interacts with DHCR7. **A** Mass spectrometry assay depicted the DHCR7 peptides pulled down by anti-RTN3 antibody. **B** WB analysis of IP assays using the indicated antibodies conducted in 293 T cells transiently transfected with the indicated plasmids. **C, D** WB analysis of IP assays using RTN3 antibody conducted in KTC-1 cells (**C**) and K1 cells (**D**). **E** Schematic representation of truncated RTN3. **F** WB analysis of IP assays using the anti-FLAG antibody conducted in 293 T cells transiently transfected with the truncated RTN3 plasmids.

To elucidate the mechanism by which RTN3 influences thyroid cancer progression, immunoprecipitation assay was conducted to capture RTN3 and proteins interacting with it. Mass spectrometric analysis identified specific peptides of a cholesterol synthesis enzyme, DHCR7, in the complex (Fig. 3A). To validate their interaction, Flag-tagged RTN3 and His-tagged DHCR7 were co-expressed in 293 T cells for exogenous co-immunoprecipitation assay. Flag-tagged RTN3 was detected in anti-His immunoprecipitates and *vice visa* (Fig. 3B). To explore their relationship in physical condition, we conducted co-immunoprecipitation assay using anti-RTN3 antibody in thyroid cancer cell lines KTC-1 (Fig. 3C) and K1 (Fig. 3D), the results further indicating that there may be interaction between RTN3 and DHCR7 in thyroid cancer cells.

Next, we mapped the protein sequence mediated the interaction between RTN3 and DHCR7. RTN3 is a 236-amino acid transmembrane protein localized to the endoplasmic reticulum. To identify the specific region mediating its interaction with DHCR7, we designed five truncated variants based on its transmembrane domains (TM) and cloned it into pcDNA3.1 vector (Fig. 3E). After the truncated variants were expressed in 293 T cells, co-IP assays were conducted (Fig. 3F). The results revealed that DHCR7 was not detectable only in the precipitates of RTN3-T5, suggesting that the missing 1-66 amino acid segment (N-terminal to the first transmembrane domain) is essential for the RTN3-DHCR7 interaction.

### RTN3 destabilizes DHCR7 protein by promoting its ubiquitination

**Fig. 4:**
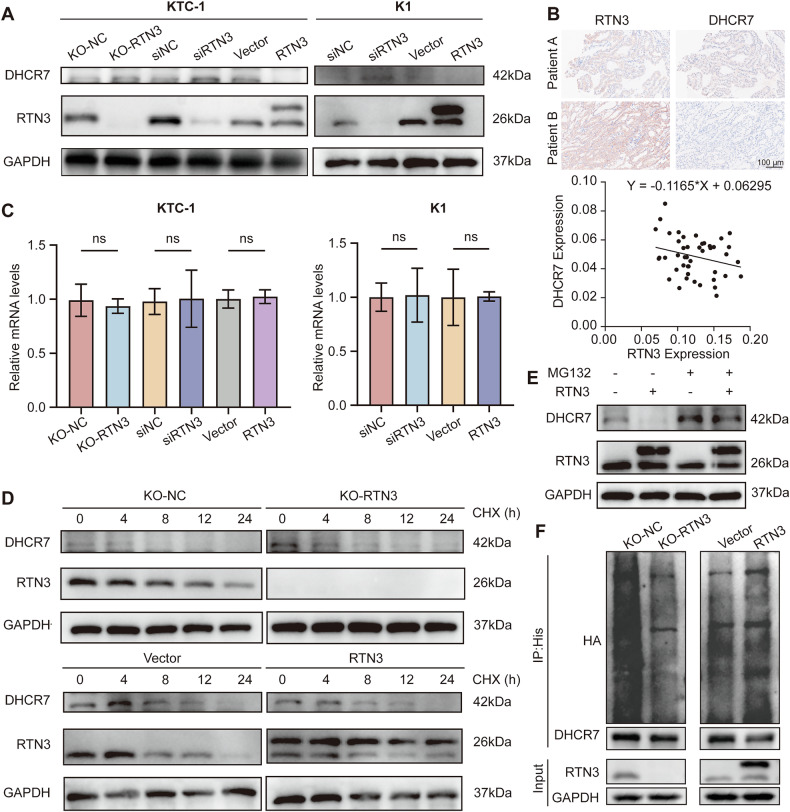
RTN3 promotes DHCR7 degradation through ubiquitination. **A** WB analysis of DHCR7 expression levels after knockout, knockdown and overexpression of RTN3 in KTC-1 (left) and K1 (right) cells. **B** IHC analysis of the correlation between RTN3 and DHCR7 protein levels of thyroid cancer tissues. Upper: Representative staining results of low and high expression of RTN3 and DHCR7; scale bar, 100 μm. Lower: Correlation analysis showed negative relationship between RTN3 and DHCR7 proteins. **C** mRNA levels of DHCR7 in RTN3 knockout, knockdown and overexpression KTC-1 (left) and K1 (right) cells. ns: *P* ≥ 0.05. **D** WB analysis of DHCR7 and RTN3 levels after CHX treatment for indicated time in RTN3 knockout (upper) or overexpression (lower) cells. **E** WB analysis of the levels of RTN3 and DHCR7 after vehicle control or MG132 treatment (20 μg/mL, 8 h) in RTN3 knockout cells. **F** Cells with RTN3 knockout or overexpression were transfected with Ub-HA plasmids and treated with MG132 (20 μg/mL, 8 h) 64 h after transfection. IP and WB assays using indicated antibodies were used to analyze the ubiquitination of DHCR7.

Following confirmation of the RTN3-DHCR7 interaction, we further investigated whether DHCR7 expression is regulated by RTN3. WB analysis revealed that DHCR7 protein levels were upregulated upon RTN3 knockdown or knockout, but downregulated upon RTN3 overexpression (Fig. 4A). IHC staining of human thyroid cancer tissue specimens two adjacent slides validated an inverse correlation between the protein expression levels of RTN3 and DHCR7 (Fig. 4B). However, qRT-PCR indicated no significant alteration in DHCR7 mRNA levels (Fig. 4C), suggesting that RTN3 modulates DHCR7 at the post-transcriptional level.

Next, we further explored how RTN3 regulates DHCR7 expression in KTC-1 cells. Cycloheximide (CHX)-chase assays demonstrated delayed DHCR7 degradation following RTN3 knockout and accelerated degradation after RTN3 overexpression, indicating RTN3 destabilizes DHCR7 (Fig. 4D). Proteasome inhibition by MG132 reversed the RTN3 overexpression-induced reduction of DHCR7 in KTC-1 cells, implying regulation via the ubiquitin-proteasome pathway (Fig. 4E). To verify the ubiquitin roles in the regulation of DHCR7 protein levels, DHCR7-His and Ub-HA were co-expressed in KTC-1 cells, followed by immunoprecipitation with anti-His antibody. Ubiquitinated DHCR7 levels showed a positive correlation with RTN3 expression in the WB assay (Fig. 4F). Taken together, these results showed that the interaction of RTN3 and DHCR7 promotes the ubiquitination of DHCR7 and therefore its degradation.

### DHCR7 plays a vital role in low RTN3-mediated thyroid cancer progression

To clarify the role of DHCR7 in thyroid carcinogenesis, we first investigated the impact of DHCR7 on tumor cell behavior. DHCR7 knockdown reduced proliferation (Supplementary Fig. S2A–C) and migration (Supplementary Fig. S2D, E), whereas overexpression of DHCR7 (using pcDNA3.1-DHCR7-His-Neo plasmid) enhanced proliferation and migration ability (Supplementary Fig. S3A–E), indicating DHCR7 is an oncogenic molecule.

**Fig. 5:**
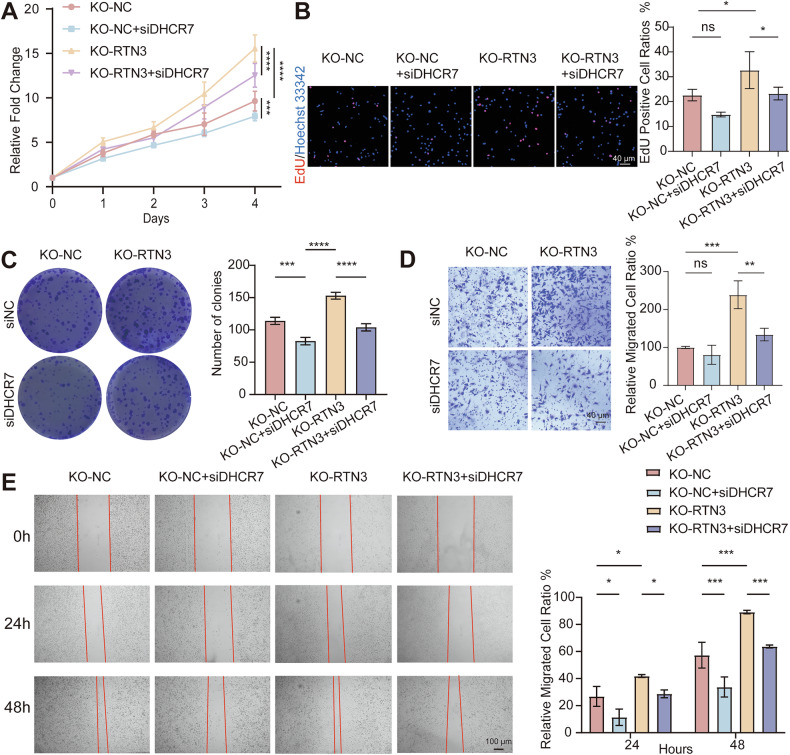
Knockdown of DHCR7 rescues the phenotypes induced by RTN3 knockout. **A–C** Cell proliferation ability determined by CCK-8 assays (**A**) and EdU assays (**B**) with representative images in the left (scale bar, 40 μm) and the quantification of EdU positive cell ratios in the right, and colony-formation assays (**C**) *n* = 3. **D**, **E** Cell migration ability determined by transwell assays (**D**) (scale bar, 40 μm) and wound healing assays (**E**) (scale bar, 100 μm), *n* = 3. *: *P* < 0.05, **: *P* < 0.01, ***: *P* < 0.001, ****: *P* < 0.0001, ns: *P* ≥ 0.05.

Rescue experiments using siRNA to knockdown DHCR7 reversed RTN3-KO-induced phenotypes. CCK-8 (Fig. 5A), EdU (Fig. 5B) and colony-formation (Fig. 5C) assays revealed markedly reduce in proliferation. Transwell (Fig. 5D) and wound healing (Fig. 5E) assays demonstrated that the enhancement of migration ability induced by RTN3 knockout was reversed. These results demonstrated that DHCR7 plays a vital role in tumor progression mediated by low expression of RTN3.

### RTN3 deactivates EGFR/ERK pathway through reducing cholesterol concentration mediated by DHCR7

**Fig. 6:**
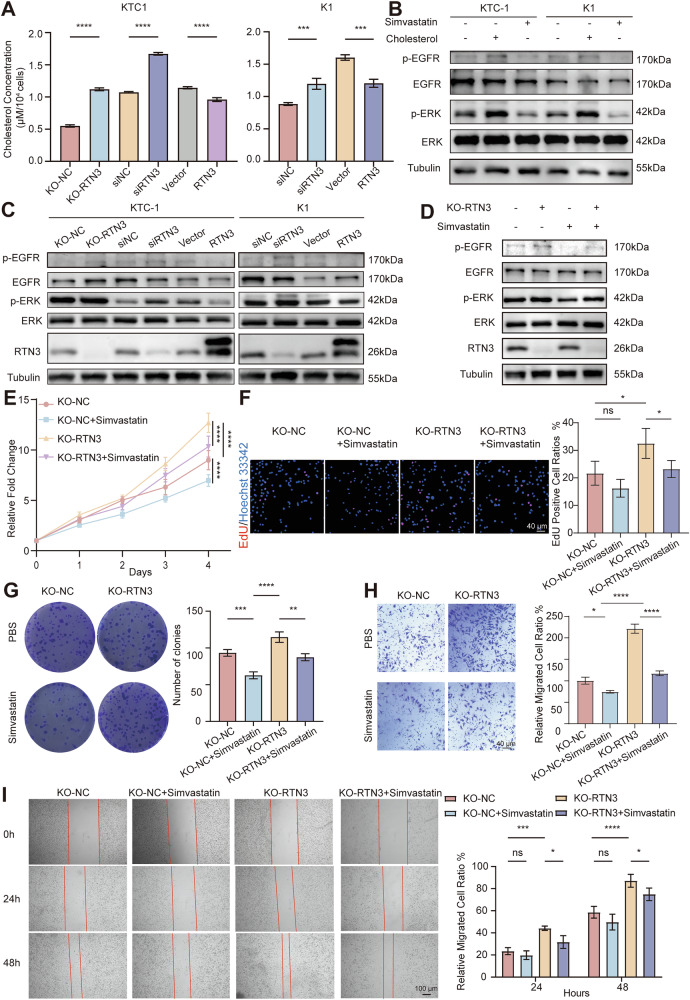
RTN3 inhibits cholesterol synthesis and therefore the activation of EGFR/ERK pathway. **A** Cholesterol concentrations in KTC-1 (left) and K1 (right) cells with RTN3 knockout, knockdown and overexpression, *n* = 3. **B** The effects of cholesterol and its inhibitor Simvastatin on the activation of EGFR/ERK pathway indicated by p-EGFR and p-ERK levels detected by WB assay in KTC-1 (left) and K1 (right) cells. **C** WB analysis of p-EGFR and p-ERK levels after RTN3 knockout, knockdown and overexpression in KTC-1 (left) and K1 (right) cells. **D** WB analysis of the effects of Simvastatin treatment on p-EGFR and p-ERK levels in RTN3 knockout cells. **E–G** Cell proliferation ability determined by CCK-8 assays (**E**) and EdU assays (**F**) with representative images in the left (scale bar, 40 μm) and the quantification of EdU positive cell ratios in the right, and colony-formation assays (**G**), *n* = 3. **H**, **I** Cell migration ability determined by transwell assays (**H**) (scale bar, 40 μm) and wound healing assays (**I**) (scale bar, 100 μm), with representative images in the left and the quantification graphs in the right, *n* = 3. *: *P* < 0.05, ***: *P* < 0.001, ****: *P* < 0.0001, ns: *P* ≥ 0.05.

Given that DHCR7 is a key enzyme in cholesterol biosynthesis and RTN3 regulates DHCR7 stability, we investigated whether RTN3 modulates cholesterol levels. Amplex Red Cholesterol assay results demonstrated increased cholesterol concentrations upon RTN3 depletion and decreased levels following RTN3 overexpression (*P* < 0.001) (Fig. 6A), paralleling DHCR7 protein levels. DHCR7 knockdown reversed cholesterol accumulation caused by RTN3 KO (Supplementary Fig. S4A), confirming RTN3 regulated cholesterol synthesis through DHCR7.

It is reported that cholesterol promotes EGFR/ERK pathway activation [31], but its role in thyroid carcinoma remains uncharacterized. To clarify this issue, we treated KTC-1 and K1 cells with cholesterol or cholesterol synthesis inhibitor Simvastatin. Figure 6B demonstrated that cholesterol elevated phosphorylated EGFR (p-EGFR) and ERK (p-ERK) levels, whereas Simvastatin reduced their phosphorylation. Considering that RTN3 downregulates cholesterol levels as validated above, p-EGFR/p-ERK levels after RTN3 knockout, knockdown or overexpression were examined. It is revealed that there is an inverse correlation between RTN3 expression and p-EGFR/p-ERK levels (Fig. 6C), and activation of the pathway caused by RTN3 knockout was rescued by both DHCR7 knockdown (Supplementary Fig. S4B) as well as Simvastatin treatment (Fig. 6D). These results confirmed that in thyroid cancer, EGFR/ERK activation is influenced by cholesterol, and that RTN3 modulates cholesterol levels via its regulation of DHCR7, thereby affecting EGFR/ERK signaling.

To further clarify the role of cholesterol in RTN3 low expressed thyroid carcinogenesis and find a possible therapy target, we treated the RTN3 knockout cells with Simvastatin. Simvastatin, which targeting cholesterol synthesis, mimicked the siDHRC7 phenotype (Fig. 6E-I). While si-DHCR7 (Fig. 5A-E) and simvastatin showed (Fig. 6E-I) limited efficacy in RTN3-wild-type cells, presumably due to low basal cholesterol, they significantly suppressed the enhanced proliferation and migration of RTN3-knockout cells, thereby rescuing the phenotype to near-normal levels. This indicated that aberrant cholesterol metabolism is likely a key driver of tumor progression following RTN3 loss.

### RTN3 low expression contributes to insensitivity to Mirdametinib in thyroid cancer

**Fig. 7:**
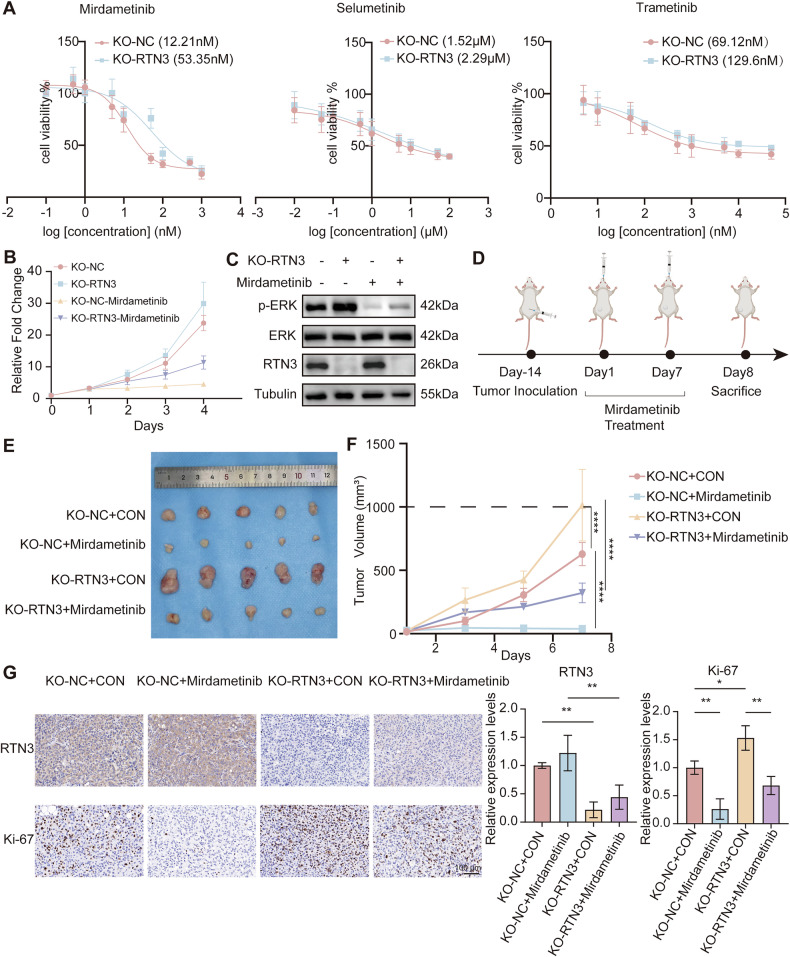
RTN3 expression levels are inversely correlated with sensitivity to Mirdametinib in thyroid cancer cells. **A** Drug sensitivity of Mirdametinib, Selumetinib, and Trametinib of KTC-1 cells and their IC50 after RTN3 knockout, *n* = 3. **B** Proliferation curve of KTC-1 cells with treatment of Mirdametinib at 20 nM. **C** The effects of Mirdametinib on the protein levels of p-ERK after RTN3 knockout. **D** The scheme of in vivo xenograft assay and Mirdametinib treatment. **E**, **F** Tumor volume (**E**) and growth curve (measured every 2 days) (**F**). **G** Representative images (left; scale bar, 100 μm) and quantification (right) of IHC staining of RTN3 and Ki-67. *n* = 5.

MEK inhibitors are one of the most used target drugs in thyroid cancer, but not all patients are sensitive to them. Since we found that low expression of RTN3 activated ERK, which is the direct downstream target of MEK, we suspected RTN3 may be a biomarker and target of Mirdametinib sensitivity. Knockout of RTN3 in KTC-1 cells reduced sensitivity to three MEK inhibitors, namely, Mirdametinib, Selumetinib, and Trametinib (Fig. 7A), and it is notable that sensitivity to Mirdametinib exhibited the most significant decrease. Therefore, we chose Mirdametinib for further study. 20 nM Mirdametinib treatment significantly impeded cell proliferation ability of KTC-1 cells (Fig. 7B). Western blotting (Fig. 7C) showed 20 nM Mirdametinib treatment efficiently suppressed ERK phosphorylation, whereas RTN3 knockout elevated phospho-ERK levels. Then we established subcutaneous xenograft models using RTN3-knockout and control KTC-1 cells, followed by Mirdametinib administration via oral gavage (Fig. 7D). Consistent with the in vitro results, Mirdametinib significantly inhibited tumor growth in control tumors, while RTN3 knockout promoted tumor progression and attenuated Mirdametinib efficacy (Fig. 7E, F). IHC confirmed successful RTN3 depletion in xenografts, and revealed that Mirdametinib downregulated Ki-67 expression significantly, whereas RTN3 knockout increased Ki-67 positive ratio (Fig. 7G). This finding demonstrated that RTN3 knockout compromises the anti-proliferative activity of Mirdametinib, suggesting a reduction in drug sensitivity.

**Fig. 8:**
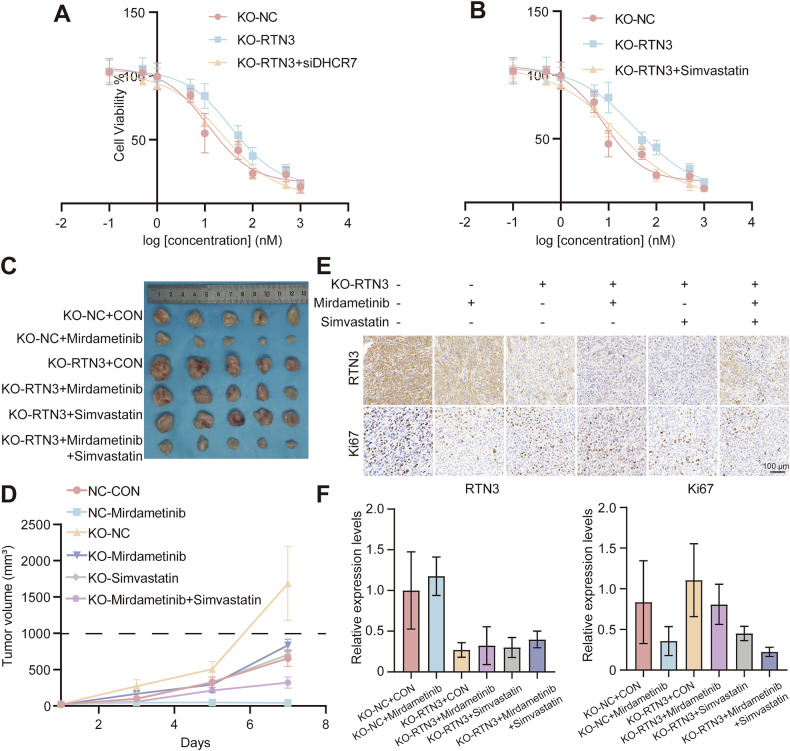
Simvastatin rescues the phenotypes induced by RTN3 knockout. **A**, **B** siDHCR7 (**A**) and Simvastatin (**B**) rescued insensitivity to Mirdametinib caused by RTN3 KO. *n* = 3. **C**, **D** Tumor volume (**C**) and growth curve (measured every 2 days) (**D**) of xenograft tumors. **E**, **F** Representative images (**E**) (scale bar, 100 μm) and quantification (**F**) of IHC staining of RTN3 and Ki-67. *n* = 5. *: *P* < 0.05, ****: *P* < 0.0001, ns: *P* ≥ 0.05.

As Simvastatin rescued proliferation and migration of thyroid cancer caused by RTN3 KO, we suspected it may be effective to solve the insensitivity to Mirdametinib as well. In vitro assays demonstrated that RTN3 knockout conferred decreased drug sensitivity and increased cell viability. However, this effect was significantly attenuated by either DHCR7 knockdown or simvastatin application, both of which reduced cell survival back to a level close to that of the control (Fig. 8A, B). Collectively, these results showed that siDHCR7 and Simvastatin can at least partially rescue the Mirdametinib insensitivity resulting from RTN3 knockout. Tumor-bearing mice were co-treated with Mirdametinib and Simvastatin, which revealed that Simvastatin effectively overcome the Mirdametinib insensitivity phenotype in mice bearing RTN3-knockout tumor formed with KTC-1 cell lines (Fig. 8C, D). IHC staining verified that Ki-67 positive ratio was reversed as well (Fig. 8E, F). These findings demonstrated that monotherapy with Mirdametinib exhibited a reduced inhibitory effect on RTN3-knockout tumors compared to the RTN3-high expression group. However, the combination of simvastatin with Mirdametinib enhanced the therapeutic efficacy, leading to a further suppression of tumor growth.

## Discussion

Aberrant cholesterol metabolism has been found to be closely associated with tumor drug resistance, making it a key target in cancer therapy. In this study, we demonstrated that low RTN3 expression correlates with poor prognosis and resistance to the MEK inhibitor Mirdametinib in thyroid cancer. RTN3 binds to DHCR7 and facilitates its degradation through ubiquitination. Consequently, reduced RTN3 expression leads to elevated DHCR7 levels, increasing cholesterol concentration and activating the EGFR/ERK pathway. Simvastatin, a cholesterol synthesis inhibitor, can reverse this effect, and its combination with Mirdametinib effectively enhances therapeutic efficacy. Our study reveals how RTN3 affects progression and drug sensitivity in thyroid cancer, offering a novel combination therapy approach.

Previous studies have revealed that RTN3 promotes apoptosis [32–34], suggesting its potential role in eliminating abnormal tumor cells. In addition, it facilitates EGFR endocytosis and therefore regulates aberrant EGFR activation in tumor cells [35]. In line with these findings, this study firstly demonstrated that RTN3 expression is significantly downregulated in thyroid cancer and correlates with poor prognosis. Drugs targeting MAPK pathway such as Trametinib and Cobimetinib have been developed and applied clinically in thyroid cancer patients, which improved patient progression-free survival [36–39]. However, drug resistance commonly occurs, due to mechanisms including MAPK pathway reactivation, activation of bypass signaling, and tumor microenvironment alterations, resulting in unsatisfactory therapeutic outcomes [40]. Consequently, elucidating drug resistance mechanisms and identifying novel combination therapy targets are crucial issues. As a MEK inhibitor, Mirdametinib suppresses the MAPK pathway, leading to the downregulation of cyclin D1 expression and consequently impeding mitotic progression. Our data from both in vivo and in vitro models demonstrate that RTN3 deletion diminishes the anti-proliferative efficacy of Mirdametinib in thyroid cancer. We propose that this attenuation is likely mediated by the subsequent promotion of cholesterol biosynthesis. Consequently, our findings suggest that co-administration with simvastatin may represent a viable combination therapy strategy.

Cholesterol metabolism is enhanced in tumor microenvironment [41] and participates in activating signaling pathways including estrogen-related receptor alpha (ERRα) and Hedgehog (Hh) [12, 13]. In addition, cholesterol modulates the function of immune cells, including CD8 + T cells and macrophages [42, 43]. Consequently, the exploration of targeting cholesterol metabolic processes emerged in cancer treatment, aiming to offer novel therapeutic strategies. Previous studies have demonstrated that cholesterol promotes EGFR and Src recruiting in lipid rafts and therefore the activation of them and downstream signaling pathways [44, 45], thereby reducing tumor sensitivity to tyrosine kinase inhibitors [31, 46]. Our study firstly verified the activating role of cholesterol on EGFR/ERK pathway in thyroid cancer, partially revealed how cholesterol acts in thyroid cancer. However, it should be noted that our study has not explored whether cholesterol regulates the EGFR/ERK pathway in thyroid cancer via the same mechanism, which needs to be elucidated in future work. DHCR7, the terminal enzyme in cholesterol synthesis, plays a pivotal role in cellular metabolism that regulates Hedgehog and Wnt pathway activation, ferroptosis, and innate immunity [47–49]. Consistently, its overexpression has been reported in various cancers, including breast, ovarian, and bladder cancers, where it contributes to disease progression [50, 51]. Genome-wide association studies indicated that single nucleotide polymorphisms affecting the synthesis, mutation, and degradation of DHCR7 are correlated with the advancement of gastric and thyroid cancers [52, 53]. However, the upstream regulatory mechanisms governing DHCR7 activity in thyroid cancer remain poorly understood. Our study revealed that the ubiquitination level of DHCR7 is modulated by RTN3, which in turn affects intracellular cholesterol concentration and modulates EGFR/ERK pathway activation. These findings not only identify RTN3 as an upstream regulator of DHCR7 but also provide a mechanistic explanation for the established link between cholesterol metabolism and thyroid cancer progression, offering a new perspective for understanding its pathogenesis.

Statins impede tumor progression not only by influencing cholesterol metabolism, but also by regulating gut microbiota metabolism, tumor autophagy, ferroptosis, and the immune microenvironment [54–56]. Simvastatin represents a favorable clinical option due to its cost-effectiveness and additional benefits, such as cardiovascular protection. Previous studies demonstrated that Simvastatin re-sensitizes hepatocellular carcinoma to sorafenib and lung adenocarcinoma to TKIs [57, 58]. However, its role in thyroid cancer is poorly understood. As we revealed that cholesterol plays a significant role in RTN3 low expression mediated thyroid cancer progression, we examined the effect of combined Mirdametinib and Simvastatin on thyroid cancer. We demonstrated that such combination produces a synergistic effect in RTN3-knockout cells, providing a novel treatment strategy for patients with low RTN3 expression who exhibit resistance to Mirdametinib. It is notable that among the three MEK inhibitors, the effect of Mirdametinib was most substantially influenced by RTN3 expression levels. This observation suggests that Mirdametinib may have the closest association with lipid metabolism, although the underlying mechanisms remain unclear and warrant further investigation.

A notable limitation of this study is that the phenotypic effects of RTN3 observed in vitro, particularly cell migration capacity, were less significant than those in vivo. Although clinical specimen analysis revealed a significant difference in lymph node metastasis rates between RTN3-high and RTN3-low groups, the biological difference measured in the wound-healing assay was modest (despite being statistically significant). We speculate that this discrepancy may be because RTN3 also plays a contributory role in tumor microenvironment. However, the specific mechanism was not explored in detail in the present study and warrants further investigation in the future.

In conclusion, this study demonstrated that low expression of RTN3 promotes thyroid cancer progression via DHCR7-mediated cholesterol synthesis and activation of EGFR/ERK pathway. Simvastatin, an effective inhibitor of cholesterol synthesis, can counteract this effect obviously. These findings not only provide a foundation for elucidating the molecular mechanisms underlying thyroid cancer progression, particularly the role of cholesterol metabolism, but also propose combining MEK inhibitors with Simvastatin as a potential novel therapeutic strategy.

## Materials and Methods

### Cell culture

All cell lines were purchased from American Type Culture Collection (USA). Human PTC cell lines TPC1 and K1 and human ATC cell line BHT101 were cultured in DMEM medium (Procell, China) with 10% fetal bovine serum (FBS; VivaCell, China). Human normal thyroid epithelial cell line Nthy-ori 3.1 and human PDTC cell line KTC-1 were cultured in RPMI 1640 medium (VivaCell, China) with 10% FBS. All these cells were cultured in a humidified incubator with 5% CO_2_ at 37 °C.

### RNA isolation and quantitative real-time polymerase chain reaction (qRT-PCR)

Total RNA was extracted using Trizol (R401-01, Vazyme) and then 1ug mRNA was reverse transcripted to cDNA using HiScript III qRT SuperMix (R323-01, Vazyme) according to the manufacturer’s protocol. qRT‒PCR was performed on BioRad CFX96 Real-Time PCR Detection System (USA) using 2×AceQ qPCR SYBR Green Master Mix (Q711-02, Vazyme). Relative RNA abundance was calculated using the standard 2-ΔΔCt method. Primers used was purchased from Sangon Biotech (China) and the sequences are detailed in Supplementary Table S2.

### Protein extraction and western blotting (WB)

RIPA (BL504A, Biosharp) contained protease inhibitors (MCE) were used to lyse cells for protein extraction. Then the total protein was heated at 95 °C with SDS, separated via 10% SDS‒PAGE gel and transferred to 0.22 µm nitrocellulose membranes. The membranes were then blocked with 5% skim milk powder, incubated with primary antibodies at 4 °C overnight, incubated with the appropriate secondary antibodies and then ECL luminescent solution (BL502B, Biosharp). The images were acquired using ChemiDoc XRS+ imaging system (BioRad, USA) and analyzed using image J software. The following antibodies were used: GAPDH (10494-1-AP, Proteintech), RTN3 (68215-1-Ig, Proteintech), DHCR7 (PA5-48204, Invitrogen), ERK (4695, CST), p-ERK (4376, CST), EGFR (2232, CST), p-EGFR (2234, CST).

### Patients and specimens

Tumor tissues and adjacent noncancerous tissues were obtained from 45 PTC patients confirmed by pathological diagnosis and received thyroid surgery at Union Hospital, Tongji Medical College, Huazhong University of Science and Technology. The tissues were embedded in paraffin for further research. The protocol was approved by Ethics Committee of Union Hospital, Tongji Medical College, Huazhong University of Science and Technology ([2025] 0424-01) and was conducted in accordance with the Declaration of Helsinki.

### Immunohistochemical (IHC) staining

Tissues were fixed with paraformaldehyde and embedded in paraffin. Tissue slides (3–5 μm) were deparaffinized and subjected to antigen retrieval. Endogenous peroxidase activity was quenched with hydrogen peroxide. After washing with PBS, the slides were blocked with goat serum. This was followed by an overnight incubation with the primary antibody and subsequent washes. The slides were then incubated with a secondary antibody for 1 h, and the signal was developed using DAB. Counterstaining was performed with hematoxylin to visualize the nuclei. The slides were scanned and randomly selected for quantitative analysis by imageJ. Antibodies used: RTN3 (68215-1-Ig, Proteintech), DHCR7 (PA5-48204, Invitrogen) and Ki-67 (GB111499, Servicebio).

### siRNA and plasmid transfection

Cells were seeded in 6-well plates and cultured until the confluence reached 80%. For siRNA transfection, lipofectamine 3000 (Invitrogen) was used, and for plasmid transfection NEOFECT DNA transfection reaction (Neofect biotech) was used. All transfection procedures were performed according to the related manufacturer’s instructions. The siRNAs were synthetized by Sangon Biotech (China) and the sequences used were shown in Supplementary Table S3. pCMV-RTN3-3xFlag-Neo plasmid was purchased from Miaoling Biotechnology and pcDNA3.1-DHCR7-His-Neo plasmid was kindly provided by Dr. Yuhan Zhao.

24 h after transfection, the mRNA was extracted for qRT-PCR. 72 h after transfection, the protein was extracted for WB assays. 72 h after transfection, cells were ready for proliferation, migration and drug sensitivity experiments.

### Colony-formation assay

1 × 103 cells were seeded in six-well plates and cultured for 14 days. Change the culture medium every 3 days. Fix the colonies with paraformaldehyde and stain with crystal violet. Count the number of colonies to evaluate colony formation ability.

### Cell viability assay

For cell growth assay, cells were seeded in 96-well plates at a density of 2000 cells/well. For drug sensitivity assay, cells were seeded 5000 cells/well and treated with indicated drugs after adherence for 72 h. Then, Cell counting kit-8 (CCK-8) solution was added and incubated at 37 °C keeping away from light for 1 h. Then, the absorbance at 450 nm was measured using a microplate reader (Thermo Fisher Scientific, USA).

### EdU proliferation assay

The assay was performed using BeyoClick™ EdU Cell Proliferation Kit with AF555 (C0075S, Beyotime) according to the manufacturer’s instructions. In brief, cells were seeded in 96-well plates at a density of 10 000 cells/well until adherence. 24 h later, the cells were incubated with EdU solution for 2 h, fixed with paraformaldehyde and incubated with Click solution for 30 min and Hoechst 33342 for 10 min. The stained cells were observed and captured with microscope (Olympus, Tokyo, Japan) and the EdU positive ratio was calculated by Image J.

### Wound healing assay

Cells were seeded in 6-well plates and cultured until the confluence reached 100%. The cells were scratched using a 1 mL pipette tip in a straight line, and the exfoliated cells were removed. The scratches were observed and captured under a microscope (Olympus, Tokyo, Japan). The migration area was calculated using image J software.

### Transwell assay

In total 2 × 10^4^ cells were seeded into the upper chamber with FBS-free medium, and the lower chamber was added with culture medium with 20% FBS. 24 h later, cells migrated into the lower chamber were fixed with paraformaldehyde, stained with crystal violet, and captured and counted under microscope (Olympus, Tokyo, Japan).

### CRISPR/Cas9-mediated gene knockout (KO)

To construct RTN3 knockout cell lines, specific sgRNA was designed (sequence detailed in Supplementary Table S4), synthesized by Sangon Biotech (China) and cloned into LentiCRISPR v2 vector. This recombinant plasmid, along with helper plasmids (psPAX2 and pMD2G), was transfected into 293 T cells to produce lentiviral particles co-expressing Cas9 protein and sgRTN3. KTC-1 cells were plated in 6-well plates at 30% confluence and transduced with the harvested lentivirus. 48 h after the transduction, cells were treated with 2 μg/mL puromycin for 4 days. Following the selection, cells were then plated in 96-well plates for monoclonal expansion. RTN3 expression of these monoclonal cell lines was detected by WB assay. Positively identified RTN3-knockout KTC-1 monoclonal cells were expanded for further study.

### In vivo xenograft assay

4-week female NCG mice were purchased from GemPharmatech (Nanjing, China) and randomly assigned to groups (*n* = 5). 2*10^7 cells transduced with different lentivirus were injected into the inguinal region of the mice to develop subcutaneous tumor xenograft models. After the tumor diameter reached 5 mm, Mirdametinib (25 mg/kg/day), Simvastatin (20 mg/kg/day) or saline were given by gavage daily for 1 week. The diameters of the tumors were measured every 2 days. Tumor volumes were calculated using the formula: (length × width^2^)/2. Mice were sacrificed after 7 days of treatment and the tumors were undergone IHC staining.

### Immunoprecipitation (IP) and mass spectrometry analysis

5 × 10^7^ cells were lysed on ice using Western and IP lysis buffer (BL1322A, Biosharp). The supernatant obtained after centrifugation at 12,000 g for 10 min was incubated with protein A/G magnetic beads (HY-K0202, MCE) and antibody or IgG at 4 °C overnight. After washing 5 times, the beads were either subjected to mass spectrometry analysis or resuspended in 1x loading buffer and heated to 95 °C for 10 min for subsequent WB analysis. The following antibodies were used: DDDDK-Tag (AE005, Abclonal), His-Tag (10001-0-AP, Proteintech), RTN3 (68215-1-Ig, Proteintech), Rabbit IgG (30000-0-AP, Proteintech), Mouse IgG (B900620, Proteintech).

### Truncated RTN3 plasmid construction and transfection

Five truncated variants of RTN3 were designed based on the transmembrane domains (TM), and corresponding amplification primers were synthesized by Sangon Biotech (China), the sequences were shown in Supplementary Table S5. Using the pCMV-RTN3-3×FLAG-Neo plasmid as a template, the full-length and five truncated fragments were amplified by PCR. The amplification products were isolated and purified via DNA gel electrophoresis. Both the pcDNA3.1 vector and the PCR-amplified gene fragments were then subjected to a double digest with NheI and KpnI restriction enzymes. The resulting linearized vector and insert fragments, which now possessed compatible cohesive ends, were purified by DNA gel electrophoresis and then were ligated using T4 DNA ligase to generate plasmids capable of expressing the respective RTN3 truncations. The constructed plasmids were subsequently transformed into E. coli for amplification, yielding a high quantity of plasmid DNA for further applications.

293 T cells were seeded in 10 cm dishes and plasmids containing truncated RTN3 sequence were transfected using NEOFECT DNA transfection reaction (Neofect biotech, China) according to the manufacturer’s instructions. 64 h later, the cells were treated with 20 μg/mL MG132 (HY-13259, MCE) for another 8 h. Then the protein was harvested for Co-IP assay.

### Cholesterol level detection

The cholesterol level was measured using Amplex Red Cholesterol and Cholesteryl Ester Assay Kit (S0211S, Beyotime) according to its manufacturer’s instructions. In brief, 1 × 10^6^ cells were lysed, and the lysate was centrifuged at 4 °C for 5 min at 12,000 g to obtain the supernatant. The total cholesterol assay working solution and standard solutions were prepared according to the manufacturer’s instructions. Standards or test samples were added to a 96-well plate followed by the addition of the working solution. The mixture was incubated at 37 °C for 30 min protected from light. A570 was measured and the cholesterol level was calculated.

### Statistical analysis

GraphPad Prism 9.0 software (GraphPad Software, San Diego, CA) was used for data analyses. Results were presented as mean ± standard deviation (SD). Student’s *t*-tests and two-way ANOVA were used to compare the difference between two or more groups. Pictures were measured by Image J software. *P* < 0.05 was considered statistically significant and statistical significance were presented as *(*P* < 0.05), **(*P* < 0.01), ***(*P* < 0.001) and ****(*P* < 0.0001).

## Supplementary information

Supplementary Tables and Figures

Original western blots

## Data Availability

The datasets used and/or analyzed during the current study are available from the corresponding author on reasonable request.
