# Supplementary material for: Declined RTN3 stabilizes DHCR7 to induce cholesterol-dependent tumor progression and MEK inhibitors insensitivity in thyroid cancer
Source: Cell Death Dis. 2026 Mar 11;17(1):306. doi: 10.1038/s41419-026-08538-y (PMC13039173; doi:10.1038/s41419-026-08538-y)
Supplement: Supplementary file 1 — Supplementary Tables and Figures [file 41419_2026_8538_MOESM1_ESM.docx]

# **Declined RTN3 stabilizes DHCR7 to induce** **cholesterol-dependent tumor progression and MEK inhibitors insensitivity in thyroid cancer**

Anwen Ren ^1,2,§^, Nan Feng ^3,4, §^, Tinglin Yang ^1, §^, Zimei Tang ^1^, Huan Liu ^1^, Yi Li ^1^, Qingyi Hu ^1^, Zihan Xi ^1^, Jiaqing Zhu ^5^, Jun Zhou ^1^, Jie Ming ^1^, Nan Liu ^2^, Tao Huang ^1,*^, Ming Xu ^1,**^

^1^ Department of Breast and Thyroid Surgery, Union Hospital, Tongji Medical College, Huazhong University of Science and Technology, Wuhan, China.

^2^ Department of Thyroid Surgery, Qilu Hospital of Shandong University, Jinan, China.

^3^ Department of Head-Neck and Thyroid Surgery, The First Affiliated Hospital of USTC, Division of Life Sciences and Medicine, University of Science and Technology of China, Hefei, China.

^4^ Department of Head-Neck and Thyroid Surgery, Anhui Provincial Cancer Hospital, Hefei, China.

^5^ First Clinical College, Tongji Medical College, Huazhong University of Science and Technology, Wuhan, China.

^§^ These authors contributed equally.

^*^Corresponding Author. Tao Huang: huangtaowh@163.com

^**^Corresponding Author. Ming Xu: mingxuwhuh@hust.edu.cn

**Supplementary Table S1**

**Supplementary Table S2**

**Supplementary Table S3**

**Supplementary Table S4**

**Supplementary Table S5**

**Supplementary Figure S1**

**Supplementary Figure S2**

**Supplementary Figure S3**

**Supplementary Figure S4**

**Supplementary Table S1. Demographic and clinicopathologic features of RTN3 high and low patients**

|  |  | RTN3 High（20） | RTN3 Low（25） | p |
| --- | --- | --- | --- | --- |
| Age (mean (SD)) |  | 41.85 (10.49) | 40.76 (12.48) | 0.757 |
| Gender (%) | Female | 15 (75.0) | 16 (64.0) | 0.64 |
|  | Male | 5 (25.0) | 9 (36.0) |  |
| BMI (mean (SD)) |  | 25.35 (3.59) | 24.95 (2.86) | 0.679 |
| Multifocality (%) | Yes | 9 (45.0) | 13 (52.0) | 0.868 |
|  | No | 11 (55.0) | 12 (48.0) |  |
| Maxmium of Diameter (mean (SD)) |  | 1.70 (1.12) | 1.64 (0.92) | 0.855 |
| Capsule Infiltration (%) | Yes | 14 (70.0) | 22 (88.0) | 0.261 |
|  | No | 6 (30.0) | 3 (12.0) |  |
| Extrathyroidal Extension (%) | Yes | 5 (25.0) | 6 (24.0) | 1 |
|  | No | 15 (75.0) | 19 (76.0) |  |
| Lymph Node Dissection (mean (SD)) |  | 20.10 (27.99) | 21.40 (19.06) | 0.854 |
| Lymph Node Metastasis (mean (SD)) |  | 4.55 (8.43) | 6.68 (6.56) | 0.345 |
| Lymph Node Ratio (mean (SD)) |  | 0.14 (0.20) | 0.33 (0.23) | **0.008** |
| Extranode Extension (%) | Yes | 0 (0.0) | 4 (16.0) | 0.178 |
|  | No | 20 (100.0) | 21 (84.0) |  |
| T Stage (%) | 1 | 9 (45.0) | 14 (56.0) | 0.697 |
|  | 2 | 6 (30.0) | 5 (20.0) |  |
|  | 3 | 5 (25.0) | 6 (24.0) |  |
| N Stage (%) | 0 | 11 (55.0) | 2 (8.0) | **0.002** |
|  | 1 | 9 (45.0) | 23 (92.0) |  |

**Supplementary Table S2. Primers for qPCR**

| **Gene** | **Forward Primer (5'-3')** | **Reverse Primer (5'-3')** |
| --- | --- | --- |
| *ACTB* | CATGTACGTTGCTATCCAGGC | CTCCTTAATGTCACGCACGAT |
| *RTN3* | CCATCCATTCAAAGCCTACCTG | CACCAACATAGGTCATCAGCC |
| *DHCR7* | GCTGCAAAATCGCAACCCAA | TCCTCGTTATAGGTGGAGTCTTG |

**Supplementary Table S3. siRNA sequence**

| **Oligonucleotide** | **Sequence (5'-3')** |
| --- | --- |
| NC | UUCUCCGAACGUGUCACGUTT |
| si*RTN3*-1 | CAGUGUCAUCAGUGUGGUUUCTT |
| si*RTN3*-2 | GGAUCUACAAGUCCGUCAUCCTT |
| si*RTN3*-3 | GUCCGUCAUCCAAGCUGUACATT |
| si*DHCR7*-1 | GGAAGUGGUUUGACUUCAATT |
| si*DHCR7*-2 | GGCGAGCGUCAUCUUCCUATT |
| si*DHCR7-*3 | GACUUCUUCUGGAACGAAATT |

**Supplementary Table S4. sgRNA sequence**

| **Oligonucleotide** | **Sequence (5'-3')** |
| --- | --- |
| sg*RTN3* | CGCGGACGGCTCGGCTCCGA |

**Supplementary Table S5. Primers for truncated RTN3 plasmid construction**

| **Gene** | **Forward Primer (5'-3')** | **Reverse Primer (5'-3')** |
| --- | --- | --- |
| *RTN3* | CTAGCTAGCGCCACCATGGCGGAGCCGTCGG | CGGGGTACCTTCTGCCTTTTTTTTGGCGA |
| RTN3 1-197 aa | CTAGCTAGCGCCACCATGGCGGAGCCGTCGG | CGGGGTACCGACAATCGGGACACTGAAAA |
| RTN3 1-152 aa | CTAGCTAGCGCCACCATGGCGGAGCCGTCGG | CGGGGTACCACGAATAATGAGTTTCAGGG |
| RTN3 1-91 aa | CTAGCTAGCGCCACCATGGCGGAGCCGTCGG | CGGGGTACCGAGAAGAGCCAGGATGAGGT |
| RTN3 1-67 aa | CTAGCTAGCGCCACCATGGCGGAGCCGTCGG | CGGGGTACCCGTGGTGCCAAAGACAAACC |
| RTN3 68-236 aa | CTAGCTAGCGCCACCATGCTGATCATGCTGCTTTC | CGGGGTACCTTCTGCCTTTTTTTTGGCGA |

**
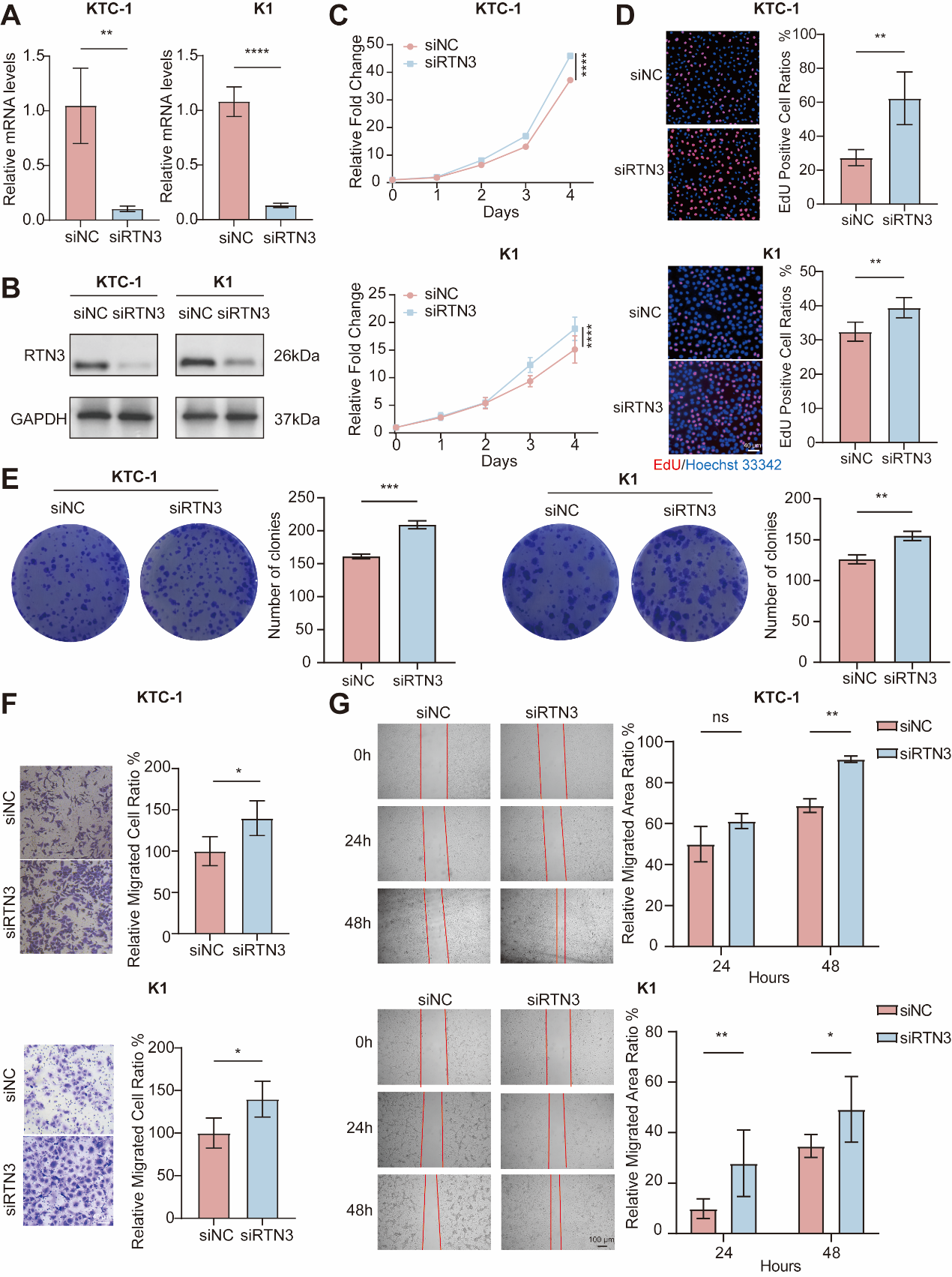
**

**Supplementary Figure S1.** Knockdown of RTN3 promotes proliferation and migration of thyroid cancer cells. **A and B,** Validation of the knockdown efficiency of siRNAs by qRT-PCR **(A)** and WB **(B)** in KTC-1 and K1 cells. **C - E**, Cell proliferation ability of RTN3 knockdown cells determined by CCK-8 assays **(C),** EdU assays **(D)** and colony-formation assays **(E)** with representative images in the left (scale bar, 40 μm) and the quantification of EdU positive cell ratios in the right, n = 3. **F and G,** Cell migration ability of RTN3 knockdown cells determined by transwell assays **(F)** (scale bar, 40 μm) and wound healing assays **(G)** (scale bar, 100 μm), with representative images in the left and the quantification graphs in the right, n = 3. *: *P* < 0.05, **: *P* < 0.01, ****: *P* < 0.0001, ns: *P* ≥ 0.05.

**
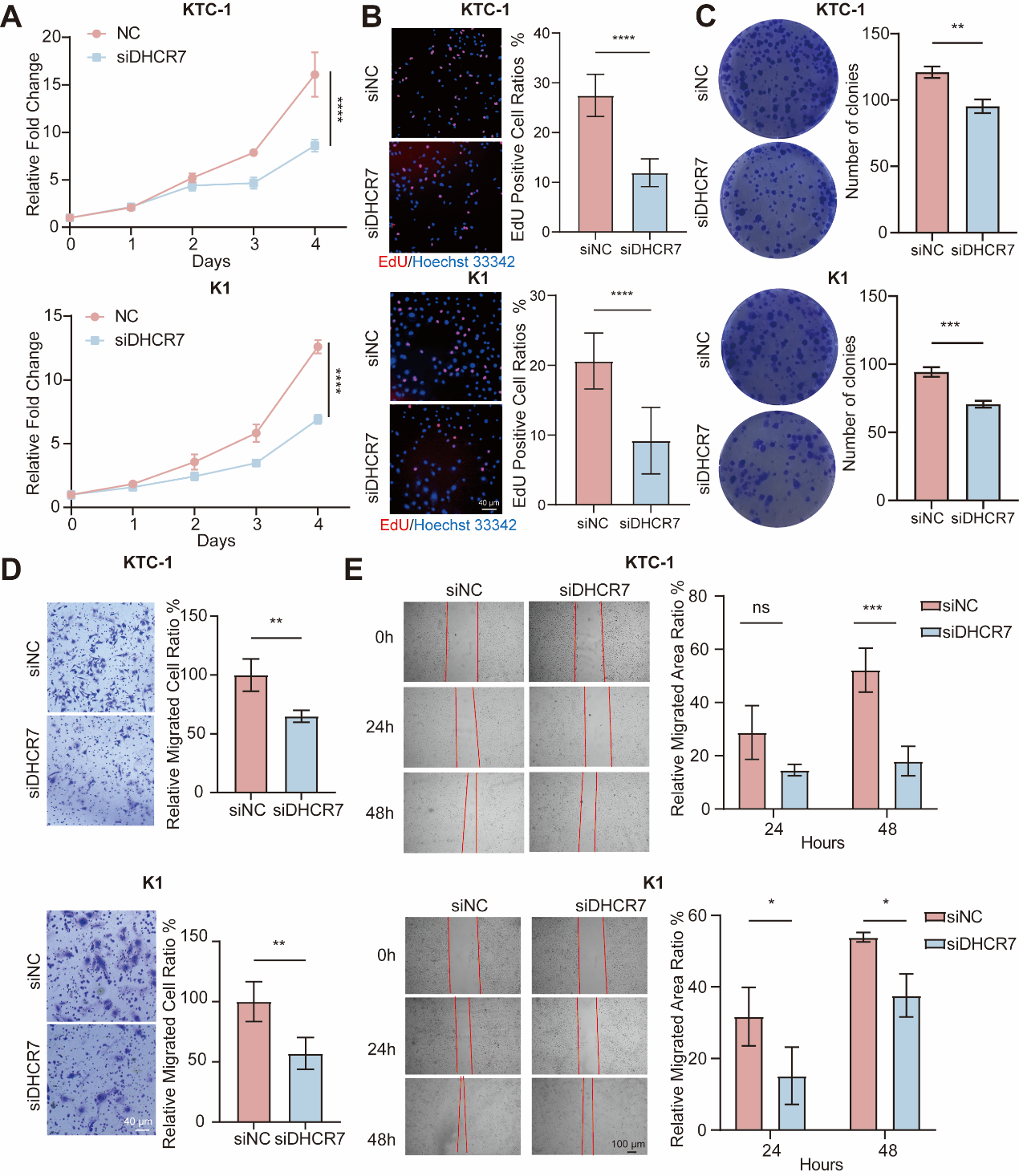
**

**Supplementary Figure S2.** Knockdown of DHCR7 inhibits proliferation and migration of cells *in vitro*. **A - C**, Cell proliferation ability of DHCR7 knockdown cells determined by CCK-8 assays **(A)**, EdU assays **(B)** with representative images in the left (scale bar, 40 μm) and the quantification of EdU positive cell ratios in the right, and colony-formation assays **(C)**, n = 3. **D and E,** Cell migration ability of DHCR7 knockdown cells determined by transwell assays **(D)** (scale bar, 40 μm) and wound healing assays **(E)** (scale bar, 100 μm), with representative images in the left and the quantification graphs in the right, n = 3. *: *P* < 0.05, **: *P* < 0.01, ***: *P* < 0.001, ****: *P* < 0.0001, ns: *P* ≥ 0.05.

**
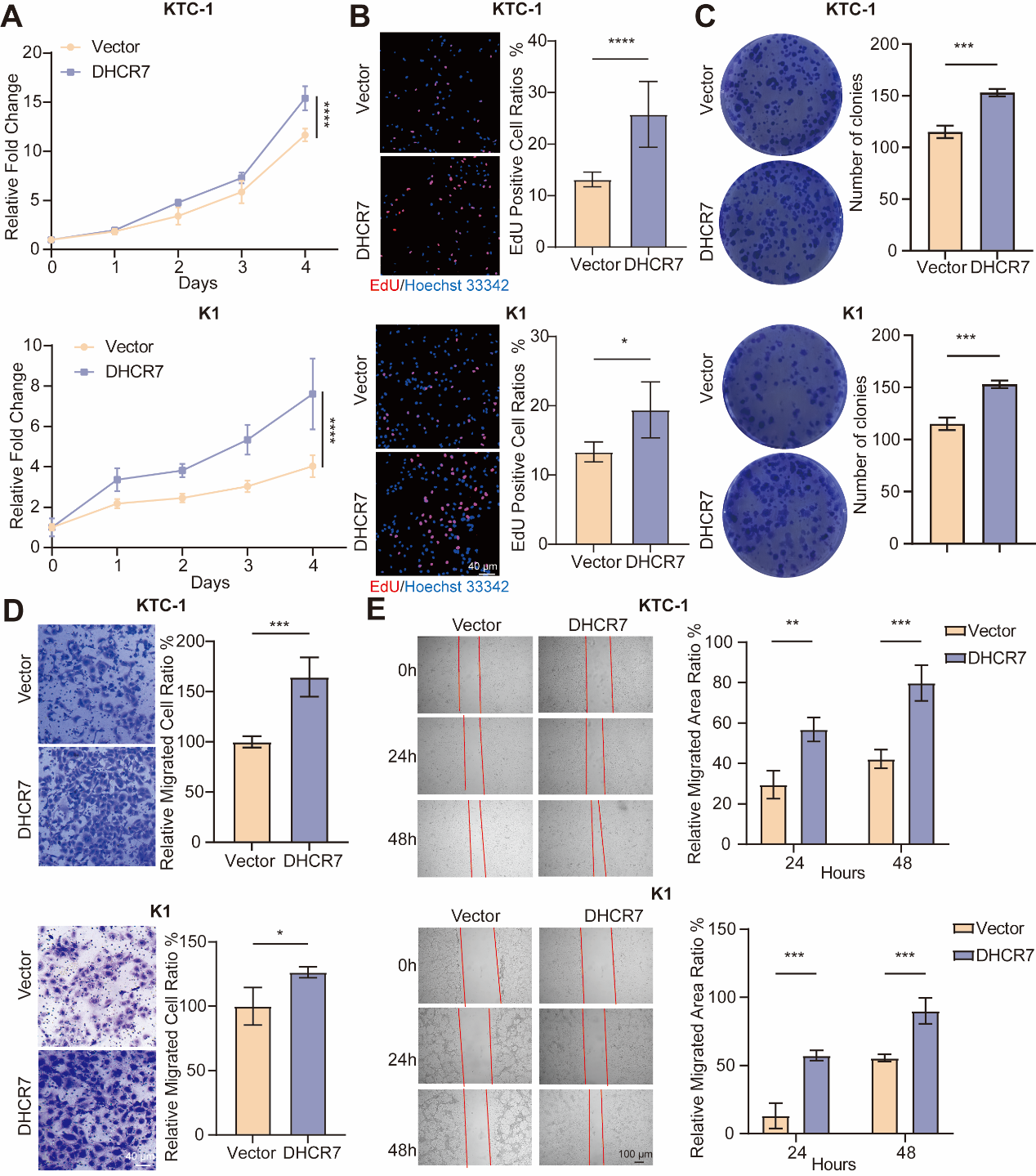
**

**Supplementary Figure S3.** Overexpression of DHCR7 promotes proliferation and migration of cells *in vitro*. **A -C**, Cell proliferation ability of DHCR7 overexpression cells determined by CCK-8 assays **(A)** and EdU assays **(B)** with representative images in the left (scale bar, 40 μm) and the quantification of EdU positive cell ratios in the right, and colony-formation assays **(C)**, n = 3. **D and E,** Cell migration ability of DHCR7 overexpression cells determined by transwell assays **(D)** (scale bar, 40 μm) and wound healing assays **(E)** (scale bar, 100 μm), with representative images in the left and the quantification graphs in the right, n = 3. *: *P* < 0.05, **: *P* < 0.01, ***: *P* < 0.001, ****: *P* < 0.0001, ns: *P* ≥ 0.05.

**
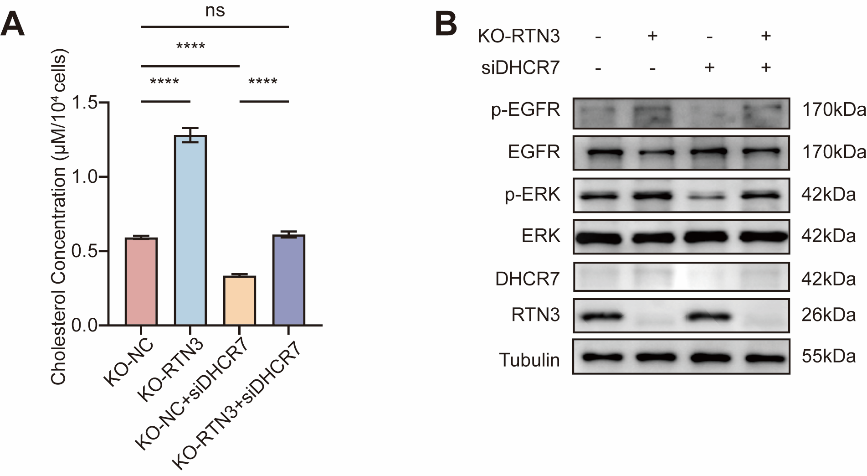
Supplementary Figure S4.** siDHCR7 reversed cholesterol accumulation and activation of the EGFR/ERK pathway. **A,** Effects of DHCR7 knockdown on cholesterol concentrations in RTN3 knockout cells, n = 3. **B,** WB analysis of the effects of siDHCR7 treatment on p-EGFR and p-ERK levels in RTN3 knockout cells. n = 3, ****: *P* < 0.0001.
