# Supplementary figures and images for: Declined RTN3 stabilizes DHCR7 to induce cholesterol-dependent tumor progression and MEK inhibitors insensitivity in thyroid cancer
Source: Cell Death Dis. 2026 Mar 11;17(1):306. doi: 10.1038/s41419-026-08538-y (PMC13039173; doi:10.1038/s41419-026-08538-y)

Figure 1


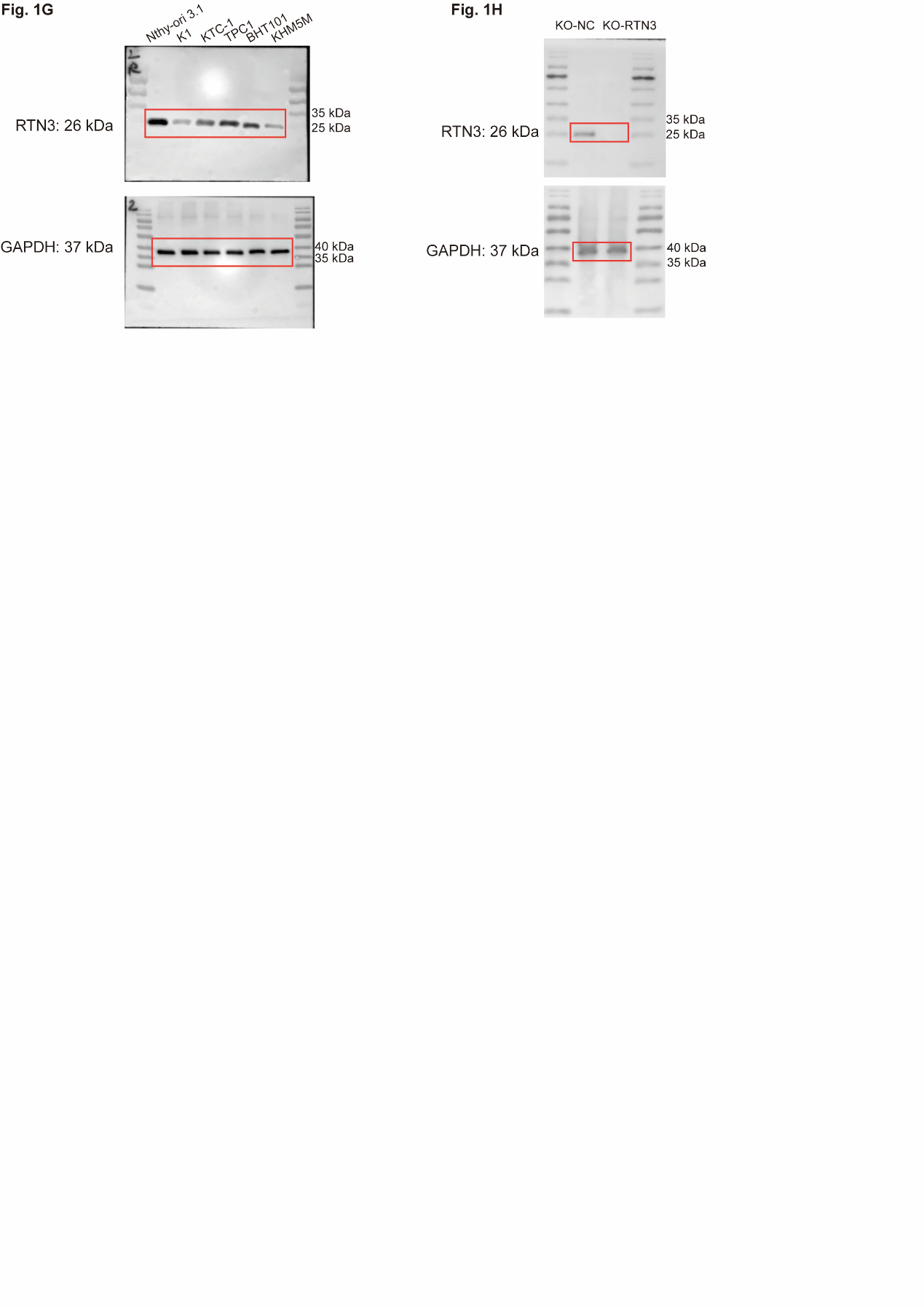


Figure 2


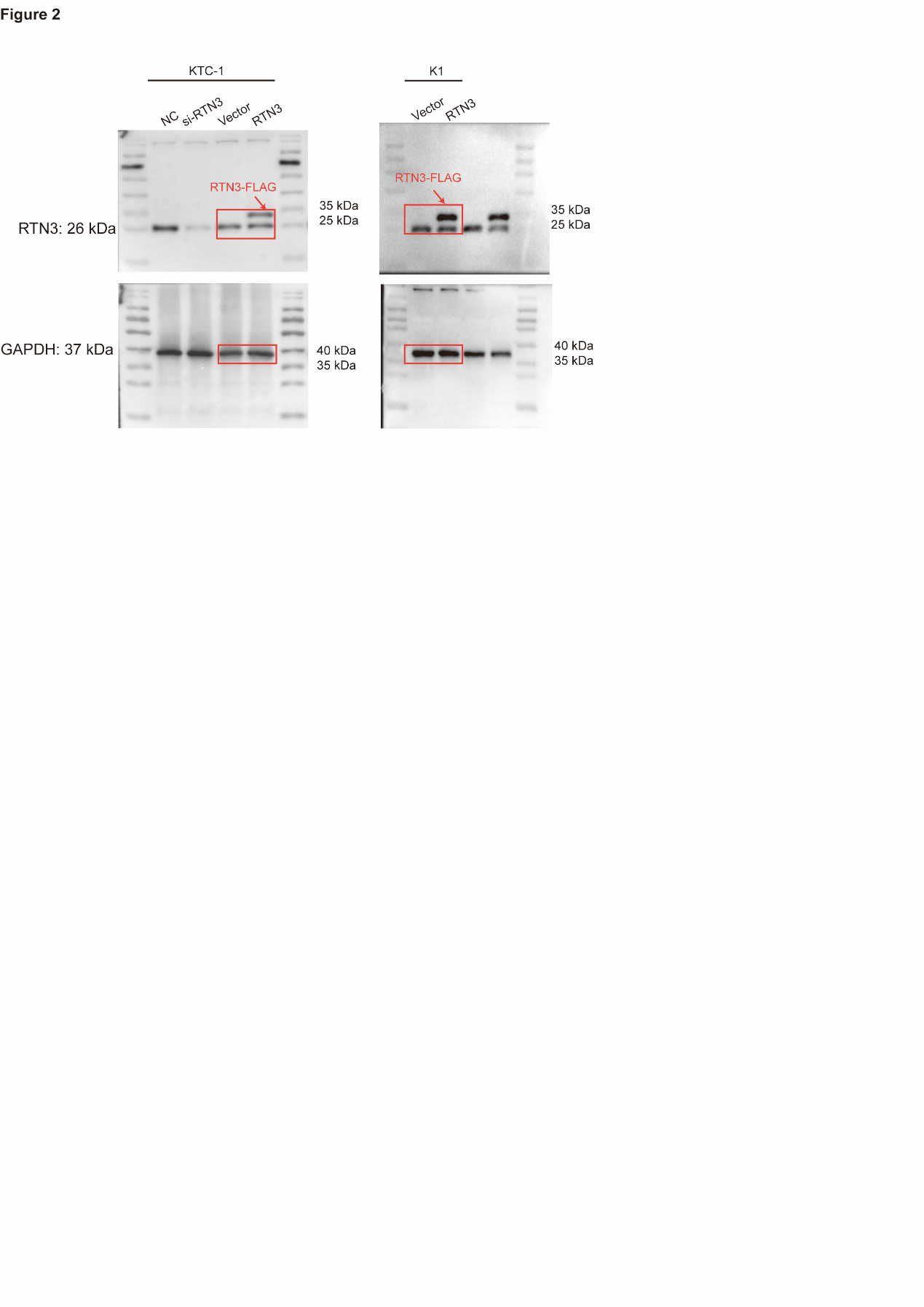


Figure 3


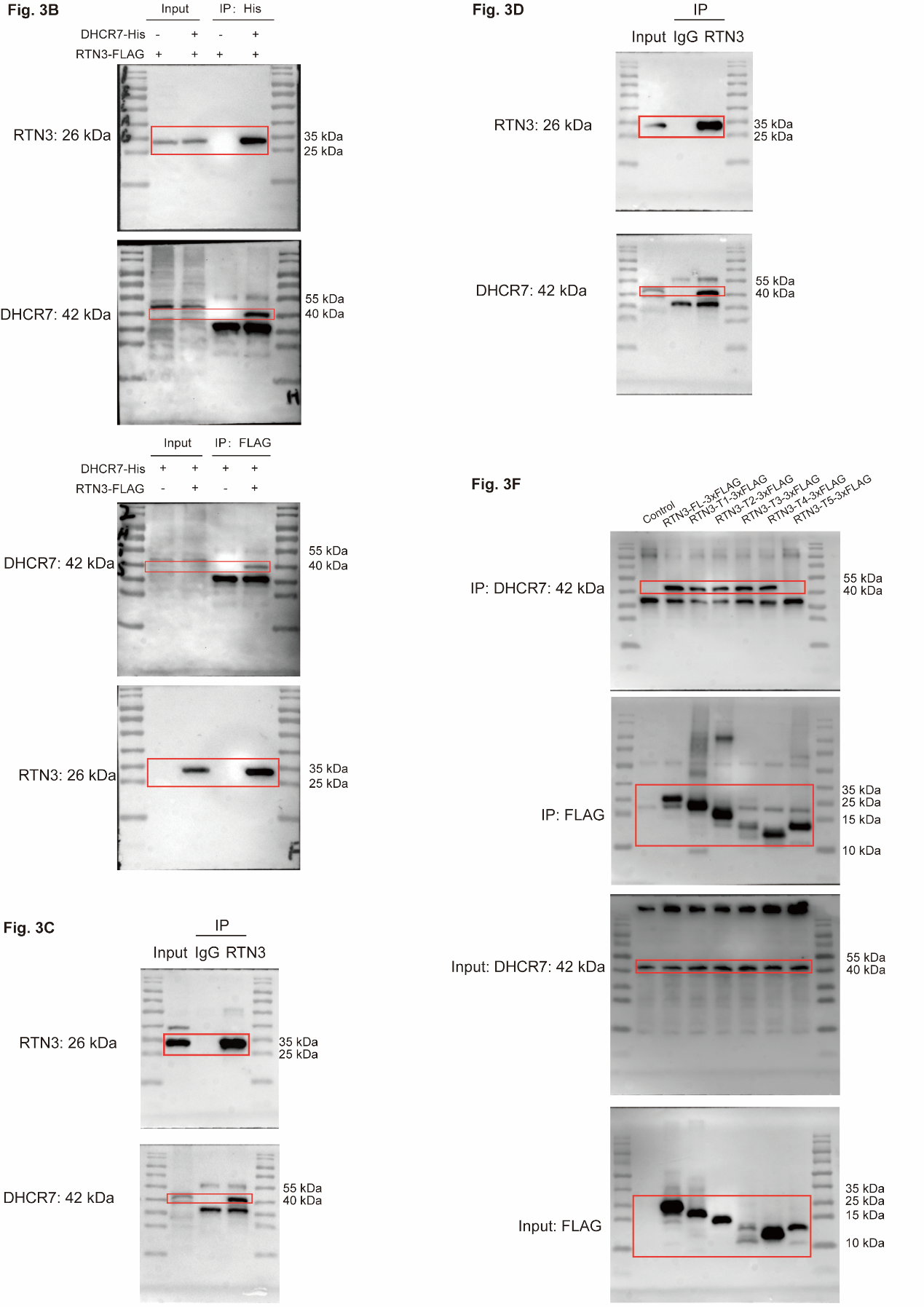


Figure 4


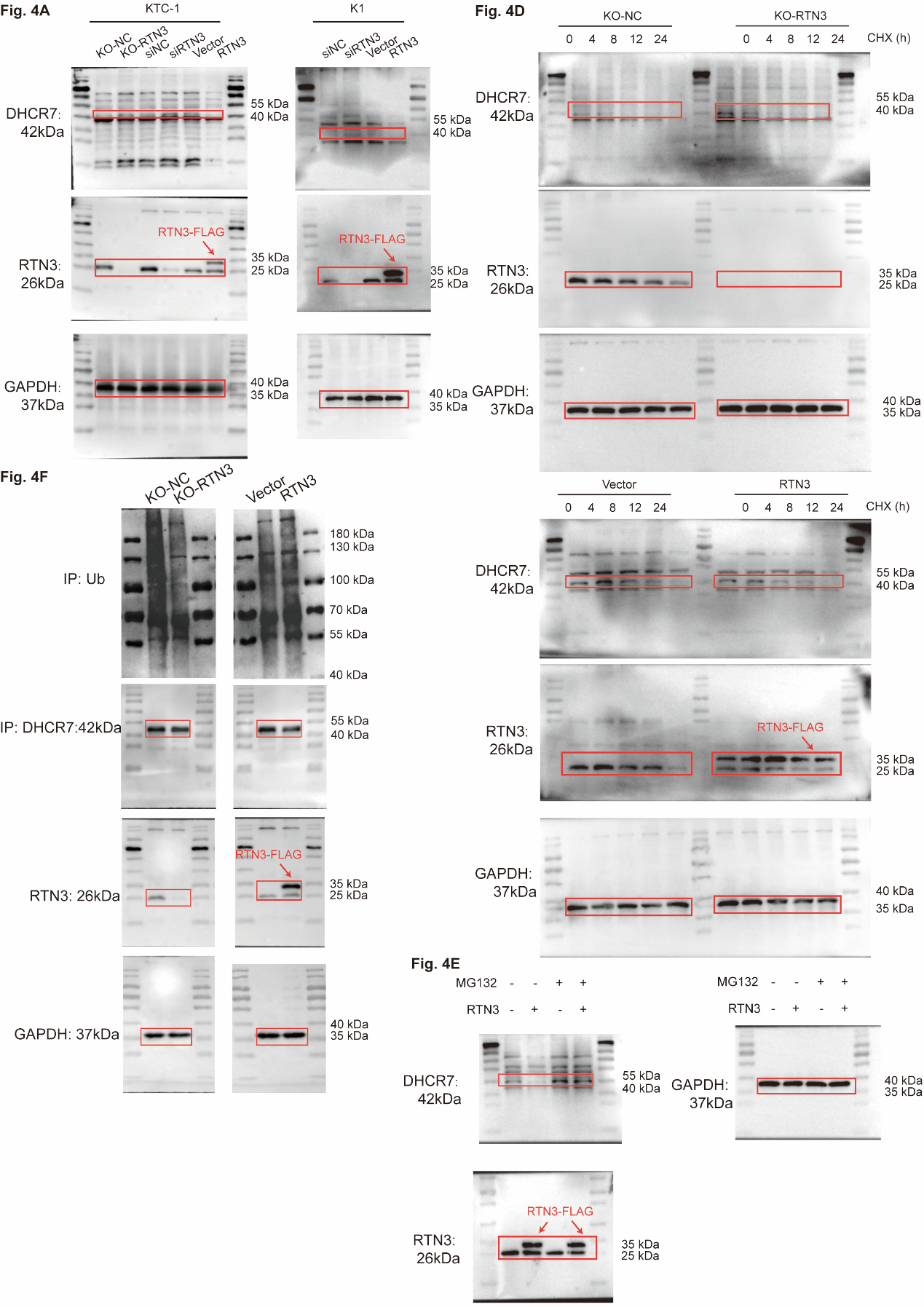


Figure 6-1


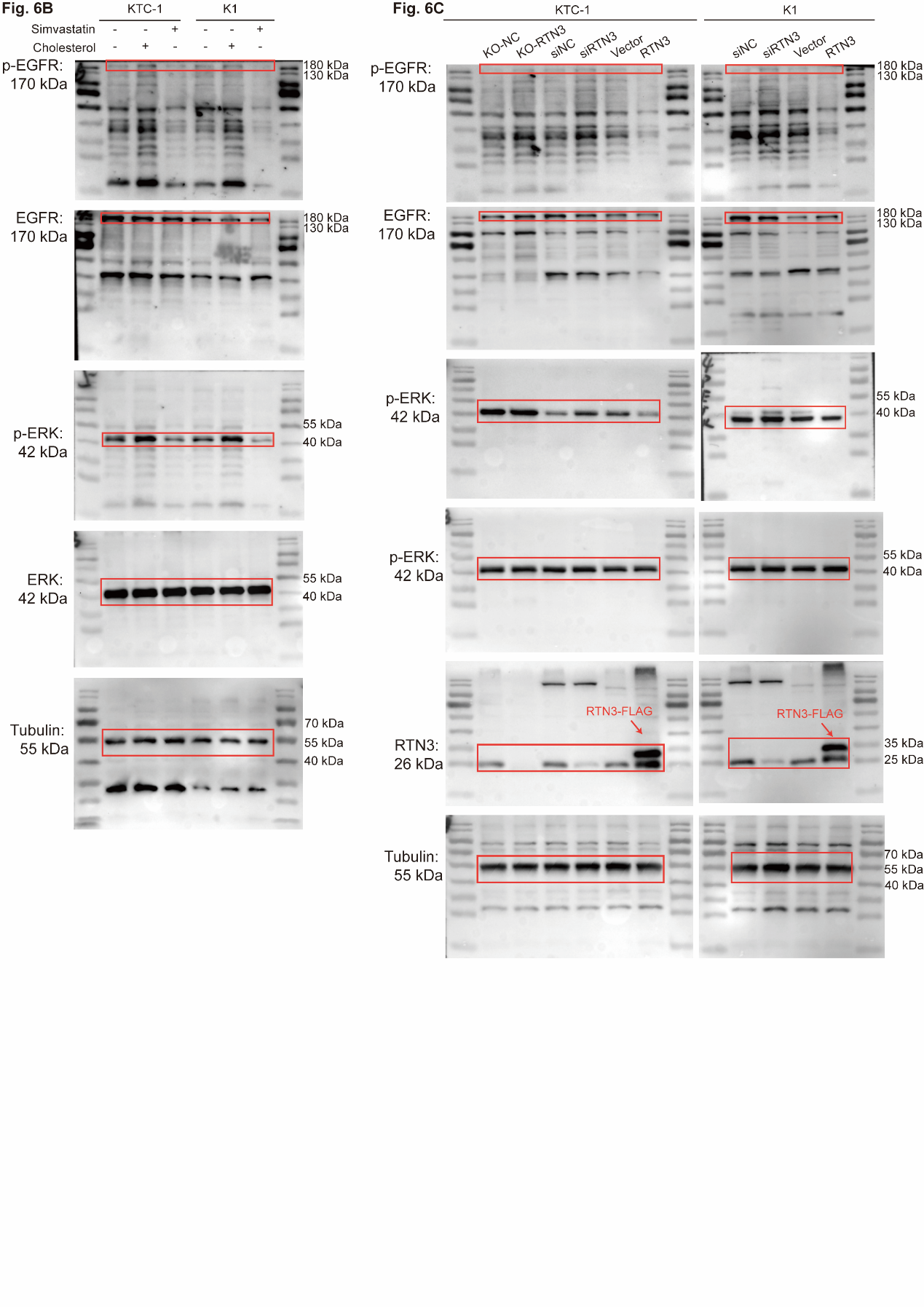


Figure 6-2


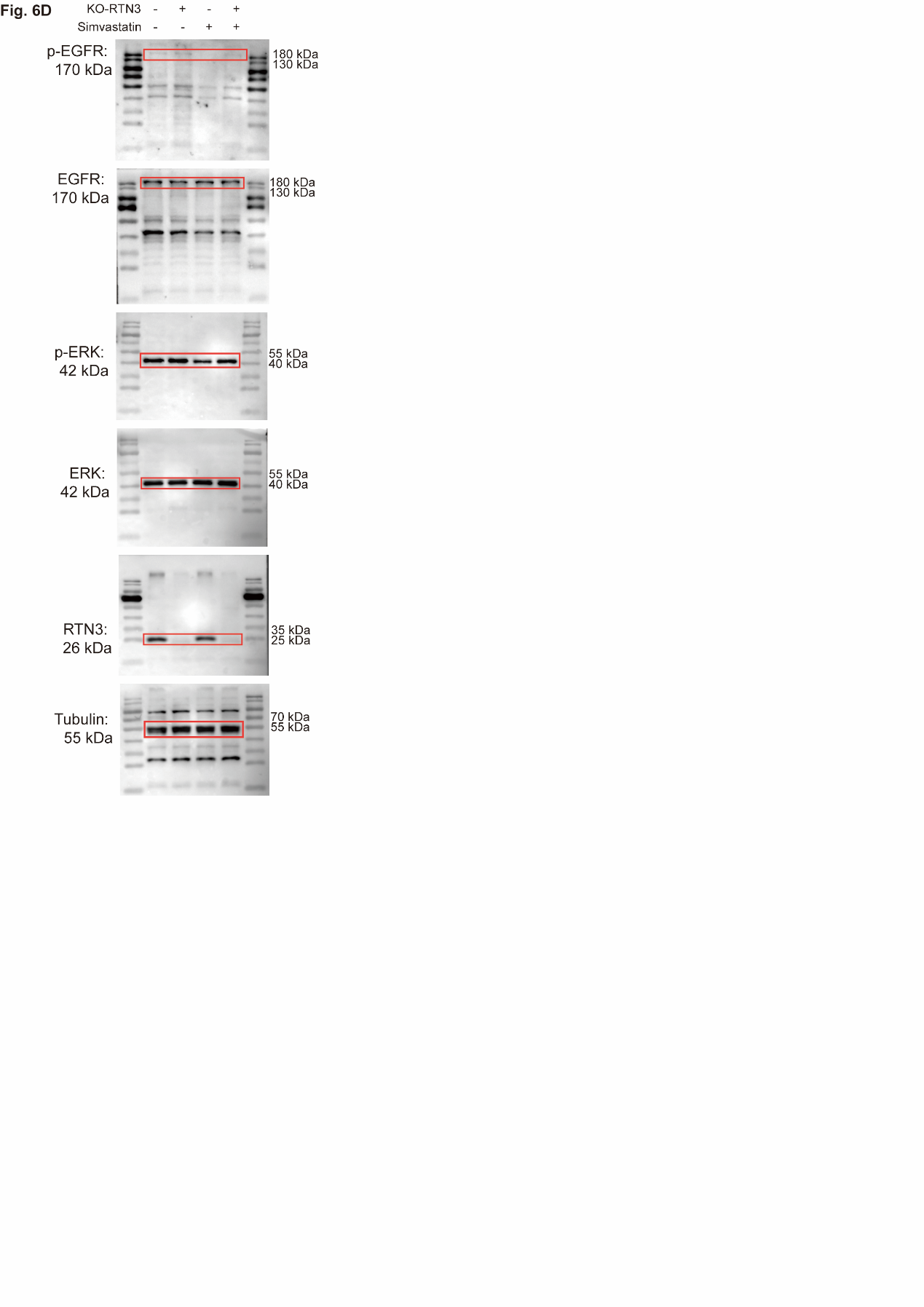


Figure 7


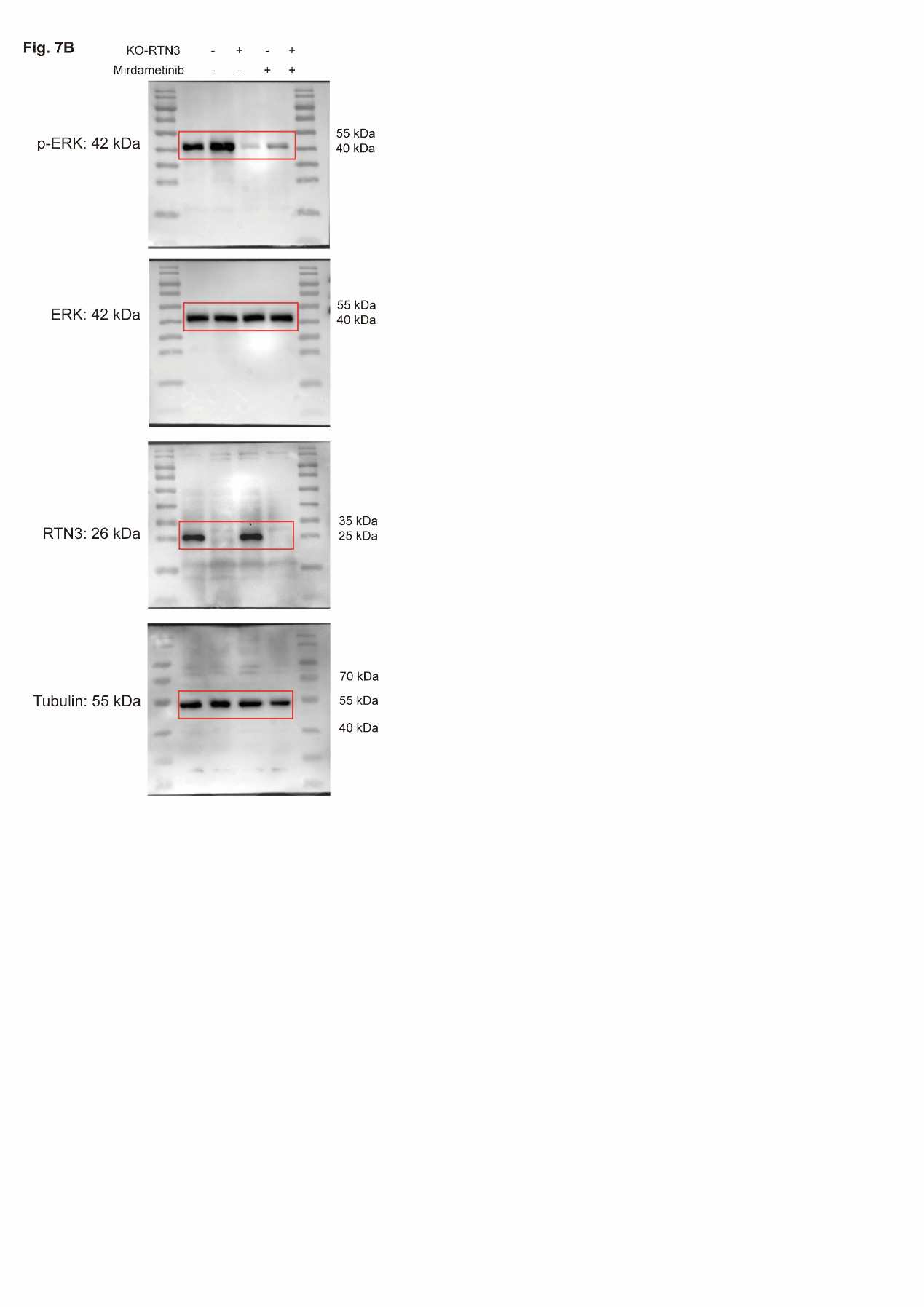


Supplementary Figures


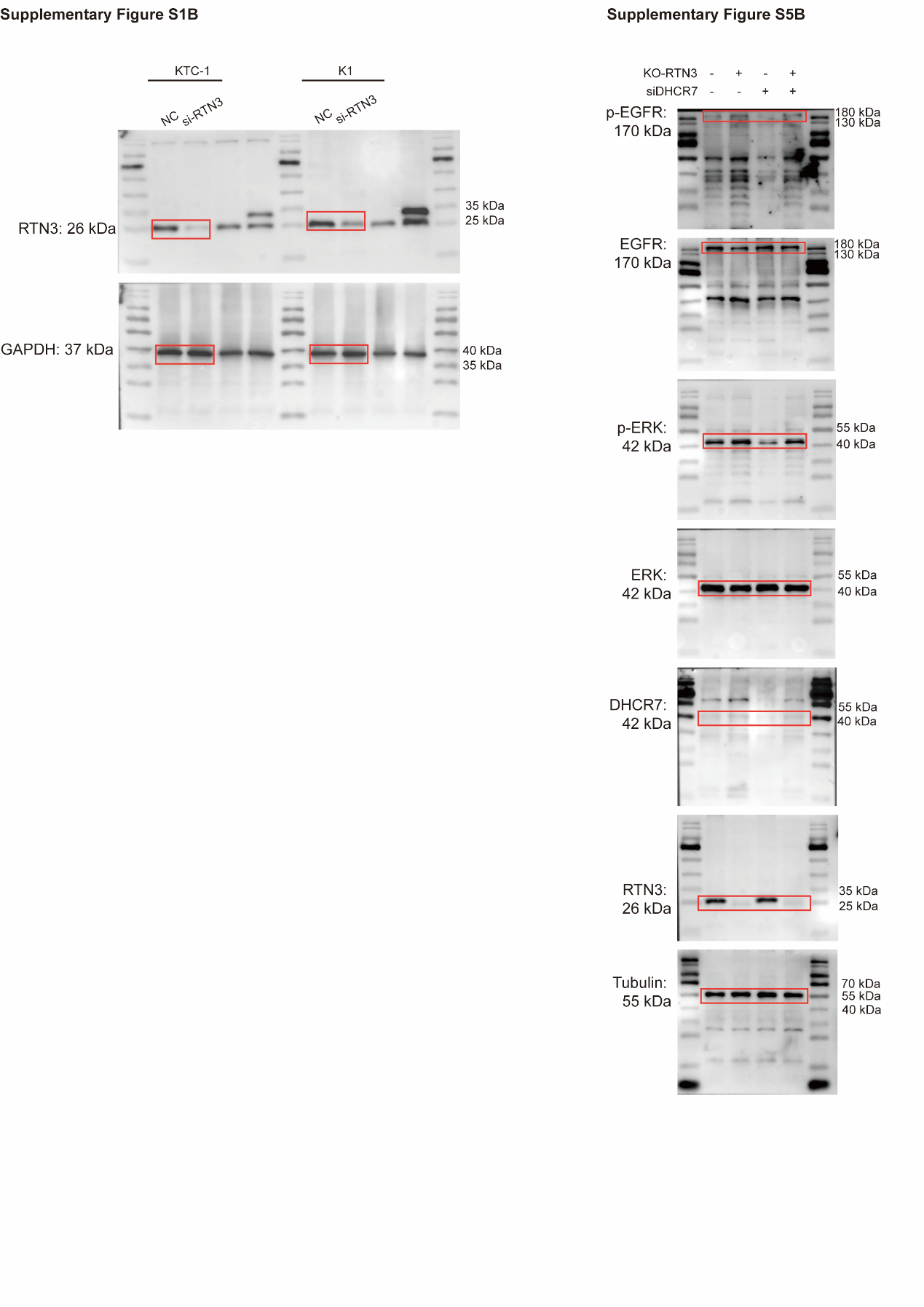

Supplement: Supplementary file 2 — Original western blots [file 41419_2026_8538_MOESM2_ESM.docx]
